# Targeting cyclic di-AMP signaling through diadenylate cyclase inhibition reduces methicillin resistance in clinical MRSA isolates

**DOI:** 10.1016/j.crmicr.2026.100615

**Published:** 2026-05-25

**Authors:** Niti Kumari, Itishree Jali, Priyanka Garg, Repally Ayanna, Vinay Bhaskar, Vasundhra Bhandari, Shailesh Sharma, Bappaditya Dey

**Affiliations:** a BRIC-National Institute of Animal Biotechnology, Hyderabad, Telangana, India; b Regional Centre for Biotechnology, Faridabad, Haryana, India; c Department of Chemical & Material Engineering, University of Alabama, Huntsville, Alabama, USA; d Faculty of Veterinary Medicine, University of Calgary, Calgary, Canada; e Department of Pharmacoinformatics, National Institute of Pharmaceutical Education and Research, Hyderabad, Telangana, India

**Keywords:** MRSA, Methicillin resistance, c-di-AMP, Diadenylate cyclase, Biofilm, STING pathway, Drug repurposing

## Abstract

**Background:**

Methicillin-resistant Staphylococcus aureus (MRSA) remains a major clinical challenge due to persistent β-lactam resistance and biofilm-associated tolerance. While canonical resistance mechanisms are well defined, non-canonical pathways such as cyclic di-adenosine monophosphate (c-di-AMP) signaling remain poorly understood, and no effective inhibitors have yet been shown to reduce MRSA resistance.

**Methods:**

Clinical MRSA isolates were analyzed for c-di-AMP levels, gene expression, biofilm formation, and host immune responses under sub-inhibitory methicillin exposure. Structure-based virtual screening of FDA-approved compounds was performed to identify inhibitors of diadenylate cyclase (DacA), followed by biochemical validation, antimicrobial susceptibility testing, macrophage infection assays, and cytotoxicity evaluation.

**Results:**

Methicillin exposure and biofilm growth significantly increased intracellular c-di-AMP levels via upregulation of DacA and associated signaling genes. Elevated c-di-AMP promoted bacterial persistence and induced STING-dependent immune modulation, characterized by increased IFN-β and reduced IL-1β in infected macrophages. Tropinone and eucalyptol were identified as DacA inhibitors that reduced c-di-AMP production, disrupted biofilms, and restored methicillin susceptibility, achieving up to 4–8 fold reduction in MICs. Both compounds demonstrated low cytotoxicity and favorable predicted ADME profiles.

**Conclusion:**

c-di-AMP signaling represents a key non-canonical regulator of methicillin resistance and pharmacological inhibition of DacA restores β-lactam susceptibility and represents a promising adjunctive strategy against MRSA.

## Highlights

• Cyclic-di-AMP is a non-canonical driver of methicillin resistance in MRSA.
• Sub-MIC methicillin and biofilms elevate c-di-AMP via DacA upregulation.
• Elevated c-di-AMP activates STING and skews macrophage responses.
• FDA approved drugs tropinone and eucalyptol identified as DacA inhibitors.
• DacA inhibition restores β-lactam susceptibility in MRSA.

## Graphical abstract

**Figure:**
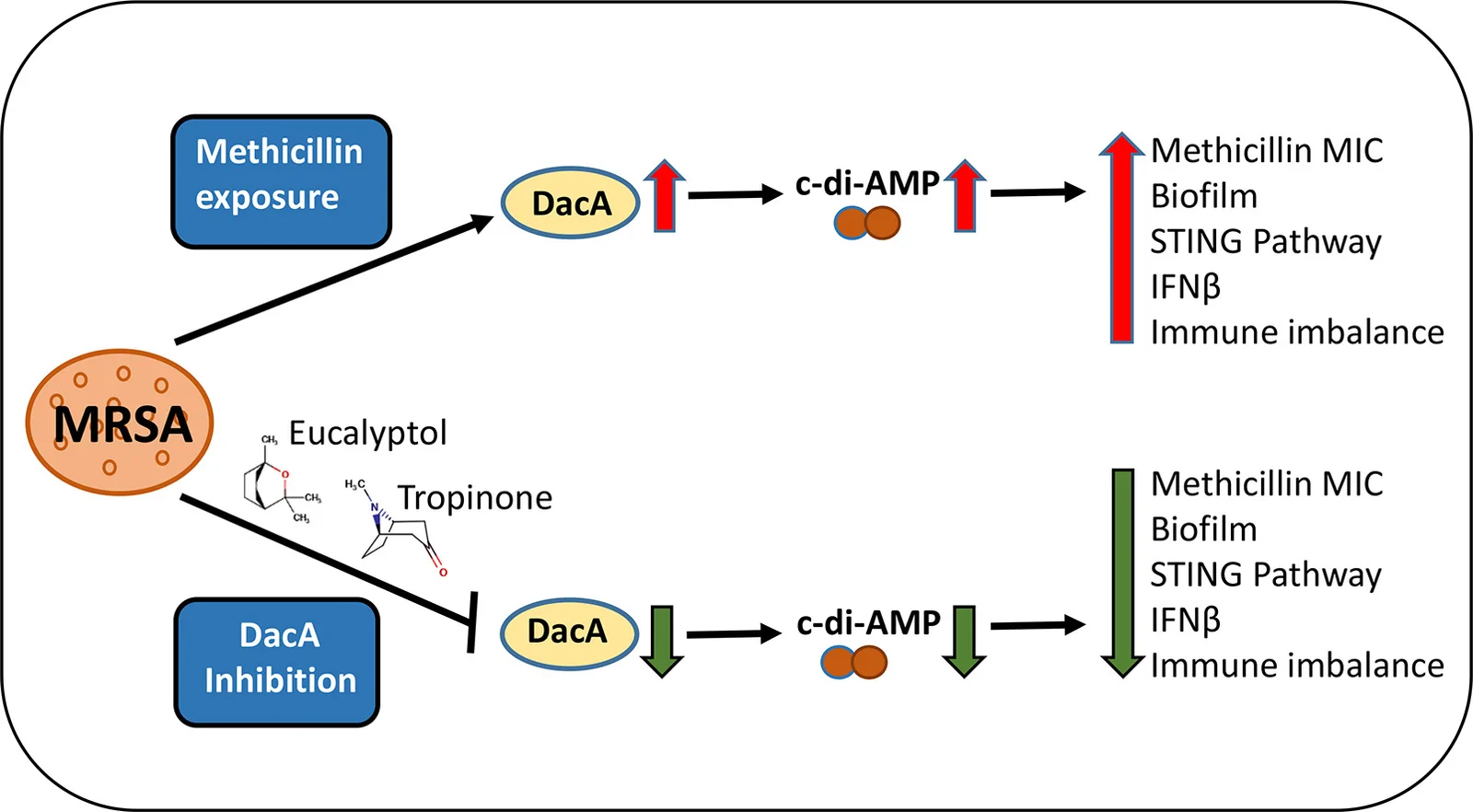

## 1 Introduction

Methicillin-resistant *Staphylococcus aureus* (MRSA) is recognized as one of the most serious bacterial threats worldwide (Collaborators, 2024). In 2021 alone, it was directly responsible for an estimated 130,000 deaths, underscoring its critical role in the global rise of antimicrobial resistance (AMR) (Collaborators, 2024). Widespread β-lactam use has selected for MRSA strains harboring low-affinity penicillin-binding proteins, conferring resistance to nearly all β-lactams (Abebe and Birhanu, 2023; Lee et al., 2018). Compounding this issue, MRSA infections are frequently associated with biofilm formation- communities of bacteria embedded in an extracellular matrix that confers increased tolerance to antibiotics and evasion of host immune defenses (Goel et al., 2021; Penesyan et al., 2021). Biofilms are implicated in 65–80% of chronic infections, including endocarditis and implanted device-related infections, underscoring the urgent need for therapeutic strategies that disrupt both resistance mechanisms and biofilm resilience (Archer et al., 2011). Recent studies have explored alternative therapeutic strategies, including nanotechnology-based antimicrobial systems and host-directed approaches, to combat resistant bacterial infections and biofilm-associated persistence (Tang et al., 2022; Zeng et al., 2026)

Bacterial second messengers regulate key physiological processes, with cyclic di-adenosine monophosphate (c-di-AMP) emerging as a critical signaling molecule in Gram-positive bacteria, including *S. aureus* (Commichau et al., 2019; Whiteley et al., 2017). c-di-AMP is synthesized by diadenylate cyclase (DacA) and degraded by specific phosphodiesterase such as GdpP (Corrigan et al., 2011) . It plays a critical role in cell wall homeostasis, osmotic stress adaptation, and potassium ion transport (Bai et al., 2014; Corrigan et al., 2011; Corrigan et al., 2015; Whiteley et al., 2017). Elevated levels of c-di-AMP have been linked to increased peptidoglycan cross-linking and resistance to β-lactams, suggesting its potential involvement in antibiotic resistance mechanisms (Corrigan et al., 2013). In addition to bacterial physiology, c-di-AMP also influences host immune responses. It is recognized by the host cytosolic DNA sensor stimulator of interferon genes (STING), leading to the induction of type I interferons such as IFN-β (Dey et al., 2015; Dey et al., 2017; Woodward et al., 2010), and inhibits NLRP3 inflammasome-mediated IL-1β release (Elmanfi et al., 2019). Such immunomodulation can shape the outcome of infection preferably by suppressing antibacterial immunity, impairing host clearance, and promoting persistent infections.

Despite critical advances, the role of c-di-AMP in methicillin resistance, biofilm formation, and host-pathogen interactions- under clinically relevant conditions, such as sub-inhibitory antibiotic exposure- remains poorly understood. Moreover, the modulation of c-di-AMP signaling, and associated immune responses during infection with clinical MRSA isolates, and potential of this pathway as a therapeutic target have yet to be systematically explored. Here, we demonstrate that pharmacological inhibition of c-di-AMP synthesis via targeting DacA, attenuates methicillin resistance, destabilize biofilms, and restore immune equilibrium in infected hosts. By integrating gene expression profiling, host-pathogen signaling, computational drug discovery, and validation via biochemical and *in vitro* infection experiments, this study explores the mechanistic ties between c-di-AMP signaling and MRSA immuno-modulation while identifying novel inhibitors with therapeutic potential. This study demonstrates that DacA inhibition enhances methicillin sensitivity in MRSA, while concurrently suppressing biofilm formation and modulating host immune responses.

## 2 Materials & methods

### 2.1 Bacterial strains and culture conditions

Clinical isolates and laboratory strains were first confirmed via streaking on HiCrome™ Universal Differential Medium and HiCrome™ Staph Agar Base. Subsequently, stocks were sub-cultured in Luria-Bertani (LB) broth at 37 °C with shaking. Glycerol stocks were prepared & stored at −80 °C. Clinical isolates were confirmed for species identity, methicillin resistance, and biofilm-forming ability by bacteriological and molecular methods.

### 2.2 Identification and characterization of *S. aureus* isolates

Clinical isolates were streaked on HiCrome™ Universal Differential Medium and HiCrome™ Staph Agar Base to confirm morphology. Species identity was verified by PCR amplification of the *DacA* gene using *S. aureus*-specific primers (Supplementary Table 1) & amplicon sequencing. PCR was performed using Taq 2x Master Mix (New England Biolabs) under standard thermal cycling conditions (95 °C 30 s; 34 cycles of 95 °C 30 s, 60 °C 60 s, 68 °C 60 s; final extension 68 °C 5 min).

#### 2.2.1 Determination of methicillin MIC by MABA

Minimum inhibitory concentrations (MICs) were determined by the microplate Alamar Blue assay (MABA) using LB broth (Elshikh et al., 2016; Tyc et al., 2016). Briefly, 100 µL bacterial suspension (∼10⁵ CFU/mL) was mixed with 100 µL serially diluted methicillin in 96-well plates and incubated at 37 °C for 3 h. Resazurin (0.02%, 20 µL) was added and fluorescence (Ex/Em 530/590 nm) measured after 3 h. MIC was defined as the lowest concentration with no color change or minimal fluorescence.

#### 2.2.2 Biofilm formation assay for S. aureus clinical isolates

Biofilm-forming capacity of methicillin-sensitive (MSSA) and methicillin-resistant (MRSA) isolates was evaluated in 24-well plates (Xu et al., 2016). Briefly, Mid-log cultures (OD₆₀₀ ≈ 0.4–0.6) were diluted to OD₆₀₀ = 0.01 in LB broth and incubated statically at 37 °C for 48 h in sterile 24-well tissue culture-treated (TC) plate. After washing with PBS (pH 7.4), biofilms were fixed with methanol, stained with 0.2% crystal violet, and solubilized in ethanol or 0.5 M acetic acid. Absorbance at 570 nm was used to quantify biomass.

### 2.3 Real-time qRT-PCR for expression analysis of c-di-AMP pathway genes

#### 2.3.1 RNA extraction and cDNA synthesis

Biofilms were grown as above, washed, and lysed using a modified TRIzol-Qiagen RNeasy method (Atshan et al., 2012). RNA purity was verified spectrophotometrically. One microgram of RNA was reverse transcribed using PrimeScript™ cDNA Synthesis Kit (Takara).

#### 2.3.2 Reverse transcription and qPCR

qPCR was performed using TB Green® Premix Ex Taq™ II (Takara) on a Bio-Rad CFX96 system. Gene targets included *cdaA, gdpP, ktrA, cpaA, kimA, trkA, pstA, nrdR, kdpD, pycA, glmM, ybbR,* and *darA,* with *rpoB* as reference. Primers are listed in the Supplementary Table 1. Cycling: 95 °C 30 s; 40 cycles of 95 °C 5 s, 60 °C 30 s; 65 °C 5 s extension.

### 2.4 Quantification of intra-bacterial c-di-AMP levels

#### 2.4.1 Extraction of c-di-AMP from planktonic and biofilm culture of S. aureus

*S. aureus* cultures (OD₆₀₀ ≈ 0.5) were harvested from planktonic or 48 h biofilm cultures. Pellets were washed with PBS, resuspended in 50 mM Tris–HCl (pH 8.0), heat-treated (95 °C, 10 min), and sonicated. Lysates were centrifuged and supernatants were separated for c-di-AMP quantification or stored at −80 °C.

#### 2.4.2 Quantification of c-di-AMP by elisa

c-di-AMP levels were measured using a competitive ELISA kit (Cayman Chemical). Standards and samples were incubated with tracer and antibody for 2 h, washed, developed with TMB, and read at 450 nm. Concentrations were calculated from standard curves (15.6–2000 pg/mL).

### 2.5 Macrophage infection, immunoassay, and cytokine profiling

#### 2.5.1 THP-1 cell culture, S. aureus infection and sample collection

THP-1 monocytes were differentiated with 10 ng/mL PMA for 72 h, rested overnight, and infected with CFSE-labeled *S. aureus* at MOI 1:20. After 1.5 h, extracellular bacteria were removed and cells incubated for 6 h. RNA, supernatants, and lysates were collected for qRT-PCR, ELISA, and immunoblotting.

#### 2.5.2 Macrophage gene expression analysis by qRT-PCR

Total RNA was extracted with TRIzol and reverse transcribed. qRT-PCR was performed for IFN-β and IL-1β, normalized to β-actin. Gene specific primers are listed in the Supplementary Table 1. Relative gene expression levels were calculated using the 2^−ΔΔCt method.

#### 2.5.3 Immunoblotting

Proteins were extracted in RIPA buffer, quantified, separated by SDS-PAGE, and transferred to PVDF membranes. Primary antibodies: anti-STING, anti-p-STING, anti-ISG15, anti-β-Actin (1:1000). HRP-conjugated secondary antibody (1:3000) was used for detection with chemiluminescent substrate. Chemiluminescent signals were visualized using the ChemiDoc Imaging System or iBright 1500 Imaging System (Thermo Fisher Scientific).

#### 2.5.4 Cytokine elisa

The supernatants from THP-1 cell culture were collected 6 hours’ post-infection, and the levels of IFN-β were quantified (pg/mL) using a sandwich ELISA kit (Krishgen BioSystems), following the manufacturer’s instructions.

### 2.6 Computational drug discovery

Detailed computational methods were provided in the Supplementary materials and a brief overview of provided below.

### 2.7 Docking based virtual screening of FDA approved drugs against DacA

The crystal structure of *S. aureus* DacA (PDB 6GYW) was energy-minimized and prepared using AutoDock Tools (Pettersen et al., 2004; Morris, 2009). A library of 10,614 FDA-approved drugs from DrugBank/PubChem was converted to pdbqt format and docked individually to DacA using MGLTools. Binding energies were ranked, and top hits visualized in PyMOL (Bramucci et al., 2012); interactions were analyzed with LigPlot (Wallace et al., 1995), and Schrödinger (Schrödinger Release 2023–3: Maestro, Schrödinger, LLC, New York, NY, 2023). Additionally, the screening capability of the docking model was assessed via receiver operating characteristic (ROC) curve analysis (Landrum, 2016; Triballeau et al., 2005). Details on the docking methodology are provided in the Supplementary Methods.

#### 2.7.1 ADME/toxicity prediction

SMILES strings of selected compounds were evaluated for ADMET properties using pkCSM, SwissADME, and ProTox-II. Favorable candidates were prioritized based on solubility, absorption, metabolic stability, and low predicted toxicity. Details are provided in the Supplementary Methods.

### 2.8 *In vitro* biochemical assays for drug target binding, inhibition, MIC, biofilm inhibition, and cytotoxicity

#### 2.8.1 Cloning, expression, and purification of DacA of S. aureus

*S. aureus* DacA was PCR-amplified from genomic DNA and gene specific primers (Supplementary Table 1), and cloned into pET21d. Expression was induced in *Escherichia coli* BL21(DE3) with IPTG (0.5 mM). Recombinant His-tagged DacA was purified by Ni-NTA chromatography, dialyzed, and analyzed by SDS-PAGE.

#### 2.8.2 Binding affinity determination using microscale thermophoresis (MST) assay

Binding affinities of tropinone and eucalyptol to DacA were measured using a Monolith NT.115 instrument with RED-tris-NTA–labeled protein (50 nM) (Singh et al., 2024). The compounds were procured from commercial vendors with >95% pure by HPLC analysis. Ligands (0.03–500 nM) were titrated. Thermophoresis was measured using 40% excitation power and 40% MST power, with a 20-second laser on-time followed by a 5-second cooling phase. Binding affinities (Kd) were calculated by plotting the fraction bound against the ligand concentration using NT Analysis software.

#### 2.8.3 Coralyne assay: fluorescence-based inhibition assay for DacA activity

To evaluate the inhibitory effect of tropinone and eucalyptol on the enzymatic activity of DacA, a coralyne-based fluorescence assay was performed (Opoku-Temeng and Sintim, 2016; Zhou et al., 2014). Briefly, Reaction mixtures (100 µL) were prepared in 96-well plates containing 10 µM coralyne, 300 µM ATP, 1 µM DacA protein, 3 mM potassium iodide (SSKI oral solution), and 20 µM of each test inhibitor (tropinone or eucalyptol) in reaction buffer composed of 40 mM Tris–HCl (pH 7.5), 100 mM NaCl, and 10 mM MgCl₂. Control wells without inhibitors served as negative control. Plates were incubated at 30 °C for 30 min, and fluorescence (Ex 420 nm/Em 475 nm) was recorded. DacA inhibition (%) = [(Control − Treated)/Control] × 100.

#### 2.8.4 Checkerboard MABA to assess combinatorial drug activity

To assess whether methicillin enhances the antibacterial activity of tropinone and eucalyptol, individual MICs were first determined by MABA (16 mg/mL and 20 mg/mL, respectively). A checkerboard MABA was then performed to test combinatorial effects. Two-fold dilutions of tropinone (8–0.125 mg/mL) and eucalyptol (10–0.15 mg/mL) were prepared below their MICs and combined with methicillin (64–8 µg/mL). Each combination was tested in 96-well plates containing LB broth. After 3 h incubation at 37 °C, resazurin (0.02%) was added to assess viability, and fluorescence (Ex 530 nm/Em 590 nm) was measured after another 3 h using a microplate reader.

#### 2.8.5 Biofilm inhibition by DacA inhibitors with or without methicillin

To evaluate the effect of tropinone and eucalyptol on *S. aureus* biofilm formation, alone or combined with methicillin, a crystal violet assay was performed under sub-MIC conditions. Bacterial cultures (OD₆₀₀ = 0.01) were seeded in 24-well plates containing LB broth and treated with methicillin, tropinone, or eucalyptol at MIC, ½ MIC, and ¼ MIC, individually or in combination (½ or ¼ MIC methicillin + inhibitor). Plates were incubated statically at 37 °C for 48 h. Wells were washed, air-dried, fixed with methanol, stained with 0.2% crystal violet, and destained with 0.5 M acetic acid. Absorbance at 570 nm was recorded after subtracting media control. Biofilm reduction across treatments was compared to assess combinatorial effects.

#### 2.8.6 Determination of cytotoxicity by LDH release assay & selectivity index

Lactate dehydrogenase (LDH) release, indicative of membrane damage, was quantified using an LDH ELISA kit (KBH0838, Krishgen) following the manufacturer’s protocol. THP-1 monocytes were differentiated into macrophages using PMA and treated with test compounds ± methicillin for 48 h. LDH release in the supernatants was quantified using a colorimetric ELISA kit; absorbance was read at 450 nm. Selectivity Index (SI) was calculated as described previously (Xu et al., 2026), and details are provided in the Supplementary Methods.

### 2.9 Quantification of c-di-AMP levels following exposure of methicillin, tropinone, and eucalyptol

#### 2.9.1 In MRSA cultures

MRSA cells were grown to mid-log phase (OD₆₀₀ ≈ 0.5) in LB broth, diluted (OD₆₀₀ = 0.01), and treated with ½ or ¼ MIC of methicillin, tropinone, or eucalyptol for 3 h at 37 °C, 200 rpm. Untreated cultures served as controls. Cells were pelleted (13,000 rpm, 5 min), and c-di-AMP was extracted and quantified using a competitive ELISA kit (Cayman Chemical, 501,960) following the manufacturer’s protocol.

#### 2.9.2 In MRSA-Infected THP-1 macrophages

THP-1-derived macrophages infected with MRSA, and post-infection were treated for 6 h with sub-MIC concentrations of methicillin, tropinone, or eucalyptol. Untreated infections served as controls. After washing with PBS, cells were harvested, and intracellular c-di-AMP was extracted and measured as above.

### 2.10 Quantification of IFN-β levels in MRSA-Infected THP-1 cells by elisa

Supernatants from MRSA-infected THP-1 macrophages treated with methicillin, tropinone, or eucalyptol were collected 6 h post-infection, and IFN-β levels were quantified by ELISA.

### 2.11 Statistical analysis

Data were analyzed using GraphPad Prism 9. *t*-test or one-way ANOVA was applied for normal distributions; Mann-Whitney U test for non-normal data. *p* < 0.05 was considered statistically significant. Results are expressed as mean ± SD, unless described in the figure legends.

## 3 Results

### 3.1 Comparative assessment of methicillin susceptibility and biofilm formation in clinical isolates of *staphylococcus aureus*

To investigate the role of the c-di-AMP signaling pathway in methicillin resistance, we first conducted a comparative analysis between methicillin-resistant (*Staphylococcus aureus*, MRSA) and methicillin-sensitive (*S. aureus*, MSSA) human clinical isolates. A total of 25 human clinical isolates of *S. aureus* were screened for methicillin susceptibility using the Microplate Alamar Blue Assay (MABA). These isolates exhibited a wide range of minimum inhibitory concentrations (MICs) against methicillin, ranging from 0.5 µg/mL to 128 µg/mL. Based on standard interpretive criteria (MIC ≤ 4 µg/mL for sensitivity, and > 4 µg/mL for resistance (Brahma et al., 2022), isolates were classified as either MSSA or MRSA, respectively. For further analysis, five human isolates each with consistent sensitive or resistant MIC values were chosen from each group, making a total of 10 isolates for comparison. Details of all clinical isolates, including their MIC values, are provided in Supplementary Table 2, and a representative image of MABA-based MIC determination is shown in Supplementary Figure 1.

**Fig. 1:**
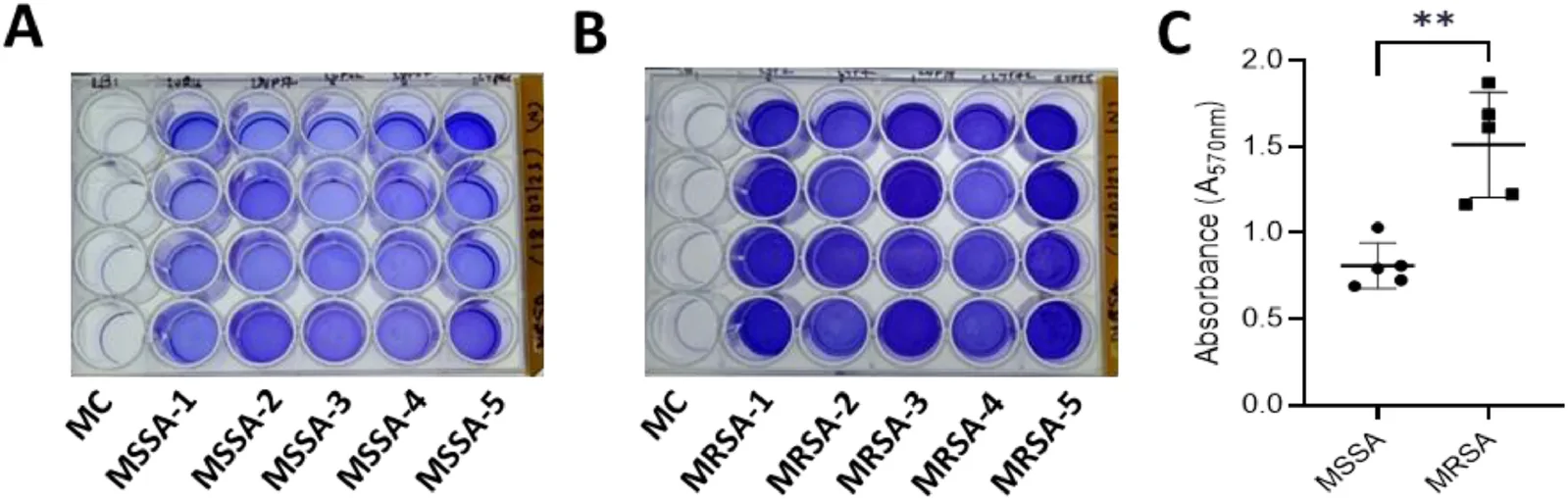
Biofilm Formation Ability of *S. aureus* Clinical Isolates. (A-B) Biofilm formation was quantified in MSSA and MRSA isolates cultured in LB media under static conditions for 48 h in 24-well polystyrene plates. Biofilm biomass was measured using a crystal violet-based assay, and (C) absorbance at 570 nm was graphically represented by scattered dot plot with mean ± SD, each dot represents average absorbance of a strain (*n* = 4 technical replicates). **p <0.01, Unpaired *t*-test (2-tailed). MC, media control.

Given that biofilm formation is a major factor contributing to phenotypic antimicrobial resistance, we next examined whether biofilm production was associated with methicillin resistance in these strains. Our results revealed that all the MRSA isolates demonstrated significantly higher biofilm biomass generation within 48 h of culture compared to MSSA isolates (Fig. 1). To further assess the relationship between methicillin resistance and biofilm phenotype, we performed a correlation analysis between MIC values and biofilm quantification (absorbance at 570 nm). The analysis revealed a positive correlation (The Spearman correlation coefficient, *r* = 0.72, *p <* *0.05*), indicating that higher methicillin resistance was significantly associated with increased biofilm production. This observation reinforces the hypothesis that biofilm formation contributes to the persistence and phenotypic resistance of MRSA strains, and may be co-regulated by pathways such as c-di-AMP signaling that is known to augment biofilm formation (Peng et al., 2016).

### 3.2 Transcriptional upregulation of c-di-AMP signaling pathway genes in mature biofilm stage in MRSA

First, to investigate the molecular basis of methicillin resistance in these clinical *S. aureus* isolates, we analyzed the presence (Supplementary Figure 2A) and expression (Supplementary Figure 2B & 2C) of selected four key genes classically associated with β-lactam resistance: *mecA, femA, blaZ*, and *fmtA*. These genes form part of the canonical resistance mechanism that enables *S. aureus* to evade the effects of β-lactam antibiotics by encoding modified penicillin-binding proteins, regulatory factors, and enzymes such as β-lactamase (Komatsuzawa et al., 1999; Lade and Kim, 2023; Zuniga et al., 2020). All four genes were present in all MRSA isolates, whereas they were absent in some of the MSSA strains (Supplementary Fig. 2A). Specifically, among the five MSSA isolates, *mecA* was absent in three, *fmtA i*n two, and *blaZ* in one, while femA was present in all MSSA isolates. Quantitative real-time PCR revealed significantly (> 2-fold) higher expression of three of the four genes in half of the MRSA isolates compared to MSSA isolates (Supplementary Figure 2B). To further assess the expression status of these genes in the MRSA isolates under antibiotic stress, we measured gene expression following sub-MIC methicillin exposure to the MRSA isolates. All four genes exhibited elevated expression in majority of MRSA isolates following methicillin treatment, however, with certain level of variability (Supplementary Figures 2C). These findings confirm that methicillin resistance in MRSA while associated with the upregulation of canonical resistance determinants, the high degree of variability in their expression across the MRSA isolates highlights a reference point for exploring additional, non-canonical mechanisms-such as those involving c-di-AMP signaling- that may further enhance survival under antibiotic stress.

**Fig. 2:**
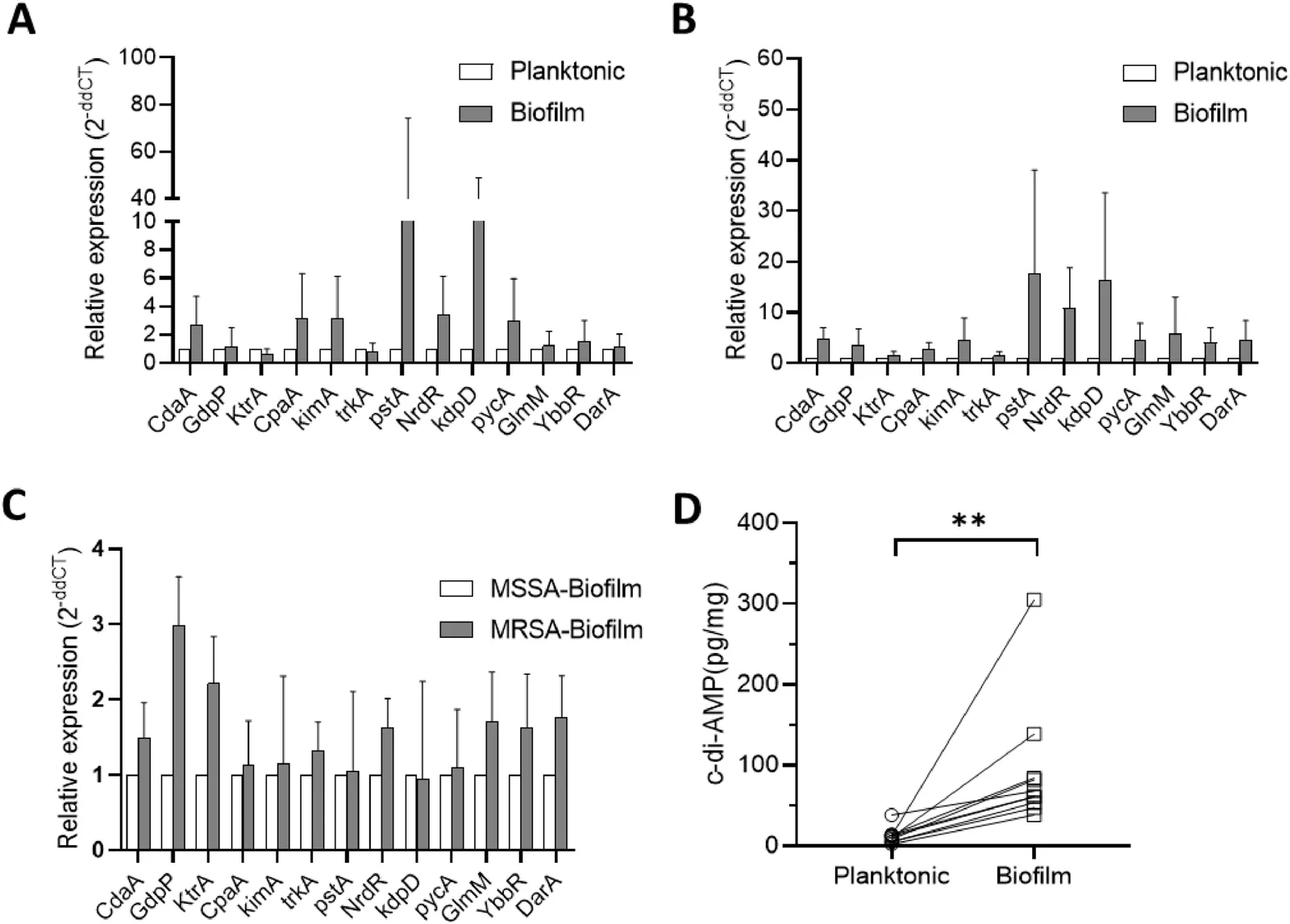
Differential Expression of c-di-AMP Signaling Pathway Genes and Intracellular c-di-AMP Levels in MSSA and MRSA Clinical Isolates. (A–C) The expression of selected c-di-AMP signaling pathway genes was analyzed in MSSA and MRSA *S. aureus* clinical isolates. RNA was extracted from planktonic cultures grown to logarithmic phase (OD₆₀₀ ≈ 0.4–0.5) and from mature biofilms (after 48 h of static incubation). Quantitative real-time PCR was used to determine fold changes in gene expression. All CT-values are first normalized to the internal control, followed by calculation of relative expression between respective groups (A). Comparative gene expression in MSSA biofilm relative to its planktonic phase. (B) Comparative gene expression in MRSA biofilm relative to its planktonic phase. (C) Relative fold induction in MRSA over MSSA biofilm states. Data represented as mean ± SD, *n* = 5 biological replicates, each with technical duplicates. (D) Intra-bacterial c-di-AMP levels are expressed as pg/mg bacterial wet weight. Data are shown as paired individual values for each strain, with planktonic phase (open circles) and biofilm phase (open squares) connected by black lines (10 strains; *n* = 3 technical replicates per strain). Statistical analysis was performed using a paired two-tailed *t*-test (**p <0.01).

Based on an extensive literature review, we selected 13 genes functionally linked to c-di-AMP signaling and downstream processes: *cdaA* (Heidemann et al., 2019)*, gdpP* (Gundlach et al., 2015)*, ktrA* (Kim et al., 2015)*, cpaA* (He et al., 2020)*, kimA* (Gundlach et al., 2019)*, trkA* (Bai et al., 2014)*, pstA* (Muller et al., 2015)*, nrdR* (Kviatkovski et al., 2024)*, kdpD* (Moscoso et al., 2016)*, pycA* (Whiteley et al., 2015)*, glmM* (Tosi et al., 2019)*, ybbR* (Bowman et al., 2016)*,* and *darA* (Gundlach et al., 2015). The key features of these genes and associated references were listed in Supplementary Table 3. Quantitative real-time PCR (qRT-PCR) was employed to compare gene expression levels in planktonic cells *versus* matured biofilms at 48-hour of growth for selected clinical isolates of *S. aureus*. As shown in Fig. 2A-B, the majority of these genes exhibited increased expression in biofilm-associated cells compared to their planktonic counterparts, indicating an activation of the c-di-AMP signaling network during biofilm development. Notably, *pstA* and *kdpD*, which encode regulators of nitrogen metabolism and potassium ion transport respectively, demonstrated the most substantial upregulation during the mature biofilm stage (Figs. 2A and 2B). Interestingly, the potassium transport-related genes *ktrA* and *trkA* showed downregulation in MSSA isolates, suggesting potential strain-specific regulation of ion homeostasis under biofilm conditions. When comparing MRSA and MSSA strains, we observed a general trend of higher expression of c-di-AMP-signaling pathway associated genes in MRSA biofilms (Fig. 2C).

To support the transcriptional data with biochemical evidence, we quantified c-di-AMP concentrations in planktonic and biofilm cultures. The results showed a significant increase in intracellular c-di-AMP levels during the mature biofilm stage across clinical isolates (Fig. 2D). This elevation reinforces the notion that c-di-AMP signaling is actively upregulated during biofilm development. Collectively, our transcriptomic and biochemical analyses underscore the importance of c-di-AMP signaling during biofilm formation and its enhanced activity in MRSA strains suggest that the c-di-AMP signaling pathway may serve as a non-canonical mechanism contributing to methicillin resistance and persistence in *S. aureus*. Unlike classical β-lactam resistance genes, this signaling network modulates multiple stress adaptation processes, and its enhanced activity in MRSA strains highlights its potential as a complementary therapeutic target alongside canonical resistance determinants.

### 3.3 Sub-MIC methicillin exposure enhances c-di-amp synthesis in MRSA *in vitro* and in host cells

**Fig. 3:**
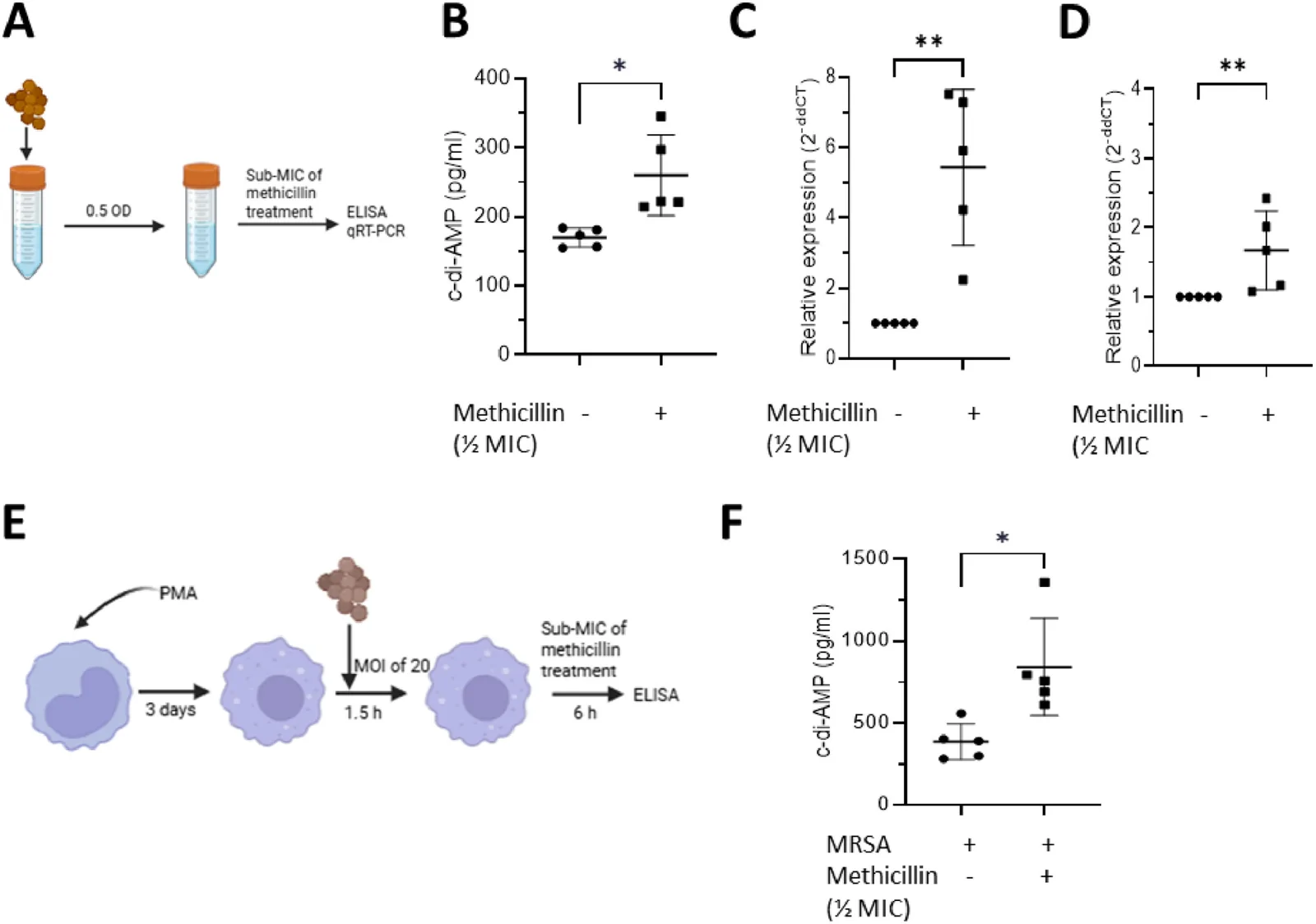
Methicillin-Induced Modulation of c-di-AMP Levels and Gene Expression in *S. aureus* and Infected Host Cells. (A) Flow chart depicting the workflow for quantification of intra-bacterial c-di-AMP levels by ELISA and gene expression analysis by qRT-PCR. (B) Intra-bacterial c-di-AMP levels were quantified in S. aureus isolates following treatment with sub-MIC concentrations of methicillin. Cultures were grown to the logarithmic phase (OD₆₀₀ ≈ 0.5), treated with sub-MIC methicillin, and incubated for 3 h. Bacterial pellets were harvested, and cellular extracts were prepared for c-di-AMP quantification using a competitive ELISA. (C–D) The expression of c-di-AMP metabolism-related genes *cdaA* and *gdpP* was evaluated under the same conditions. Total RNA was extracted after 3 h of treatment, and relative gene expression was analyzed by qRT-PCR. Fold changes were calculated relative to untreated controls. (E) Flow chart illustrating the quantification of intracellular c-di-AMP levels in host cells following MRSA infection, using ELISA. (F) THP-1-derived macrophages were infected with MRSA at a multiplicity of infection (MOI) of 1:20. At 6 h post-infection, intracellular c-di-AMP levels were quantified in MRSA treated with sub-MIC methicillin compared to untreated controls, using a competitive ELISA. Data are presented as scatter plots showing individual biological replicates with mean ± SD (*n* = 5; each with technical duplicates). Statistical analysis: unpaired two-tailed *t*-test for B and F (*p <0.05); two-tailed Mann–Whitney test for C and D (**p <0.01).

Sub-inhibitory concentrations (sub-MIC) of antibiotics represent clinically relevant stress conditions that often precede the development of resistance and biofilm adaptation. To investigate the effect of sub-MIC methicillin exposure on the c-di-AMP signaling pathway, we quantified intracellular c-di-AMP levels in these clinical *S. aureus* isolates (Fig. 3A). c-di-AMP concentrations increased in all the clinical isolates following sub-MIC concentration of methicillin treatment (Fig. 3B). To further understand the underlying regulatory mechanisms, we examined the transcriptional response of *cdaA*, the gene encoding diadenylate cyclase (DacA), which catalyzes c-di-AMP synthesis. Quantitative real-time PCR analysis revealed that *cdaA* expression was significantly upregulated in most sub-MIC methicillin-treated clinical isolates (Fig. 3C). This transcriptional upregulation was consistent with the observed increase in c-di-AMP levels, suggesting that methicillin stress induces DacA-mediated c-di-AMP synthesis as part of the bacterial adaptive response. Interestingly, we also observed an increase in the expression of *gdpP*, which encodes the phosphodiesterase responsible for c-di-AMP degradation (Fig. 3D), suggesting a feedback mechanism to regulate intracellular c-di-AMP levels under stress. These observations indicate activation of this second messenger pathway in response to antibiotic stress.

Importantly, to evaluate whether this effect persists during host infection, we infected THP-1 macrophages with MRSA in the presence or absence of sublethal methicillin exposure (Fig. 3E). ELISA-based quantification revealed a significant increase in c-di-AMP levels within intracellular MRSA under methicillin treatment compared to untreated controls (Fig. 3F). This suggests that methicillin-mediated stress enhances c-di-AMP production not only *in vitro* but also within host cells, potentially contributing to immune modulation and bacterial persistence during infection. Together, these results highlight that sub-MIC methicillin exposure leads to coordinated regulation of c-di-AMP metabolism in *S. aureus* clinical isolates, both *in vitro* and during host infection, likely contributing to stress adaptation, survival, and persistence.

### 3.4 MRSA-induced c-di-AMP activates sting signaling and modulates macrophage cytokine response

**Fig. 4:**
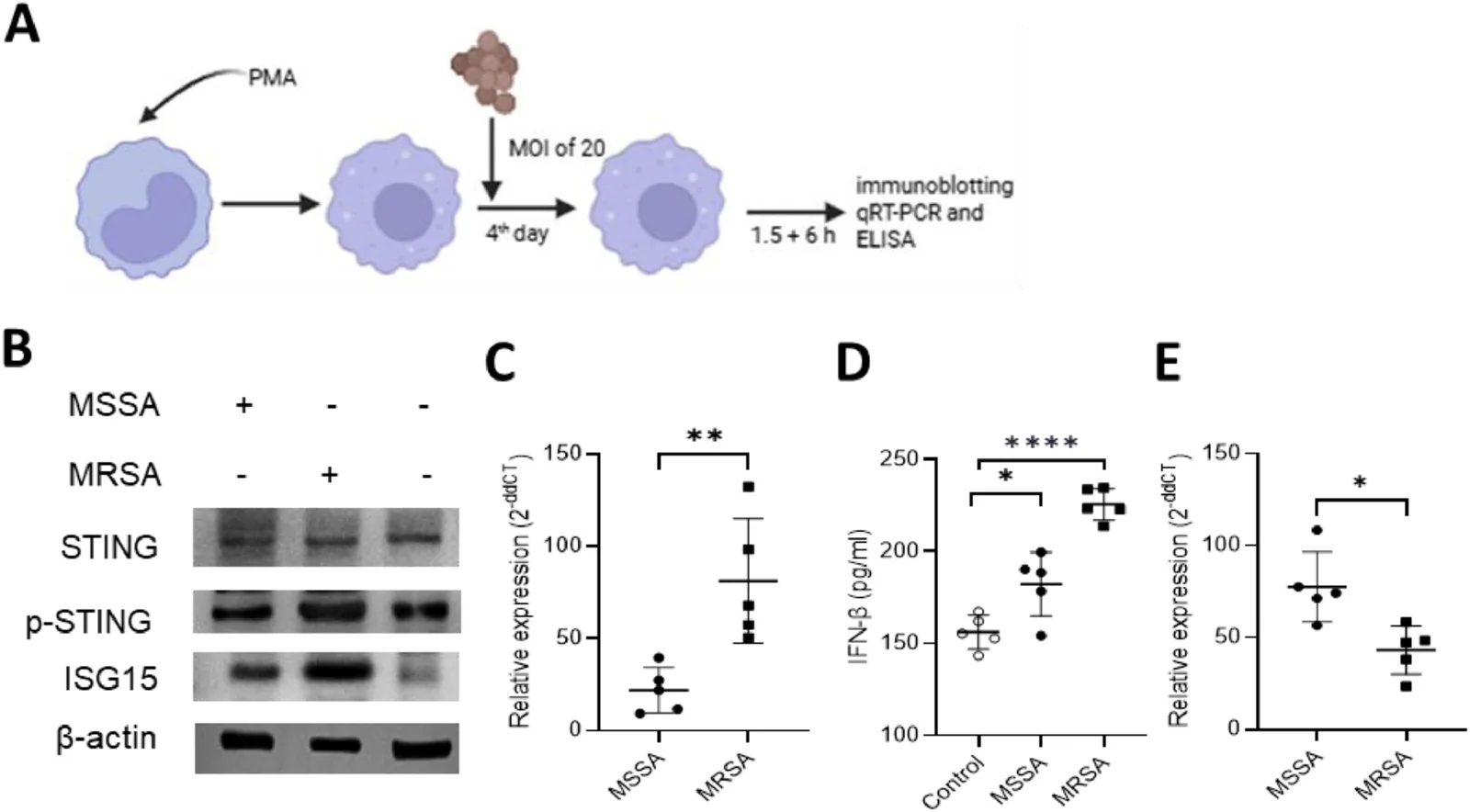
Activation of the STING Pathway, ISG15 Expression, and Cytokine Response in Macrophages Infected with MRSA or MSSA. (A) Flow chart illustrating activation of the STING pathway and cytokine responses in macrophages infected with MSSA and MRSA, followed by immunoblotting, qRT-PCR, and ELISA. (B) Western blot analysis of total STING, phosphorylated STING (p-STING), and Interferon-Stimulated Gene 15 (ISG15) in THP-1-derived macrophages infected with MRSA or MSSA. (C) Relative mRNA expression levels of IFN-β measured by qRT-PCR. Each CT value was first normalized to the internal control, followed by the calculation of relative expression with respect to uninfected cells. (D) Quantification of secreted IFN-β protein levels in cell supernatants by ELISA. (E) Relative mRNA expression levels of IL-1β (IL1B) measured by qRT-PCR. Scatter plots (C, E) depict relative expression for individual biological replicates with group mean ± SD (*n* = 5; each with technical duplicates). Statistical analysis was performed using an unpaired two-tailed *t*-test (**p < 0.01, *p < 0.05). Scatter plot (D) shows individual biological replicate values with group mean ± SD (*n* = 5; each with technical duplicates). Statistical significance was determined by one-way ANOVA followed by Dunnett’s multiple comparisons test (****p <0.0001, *p <0.05).

To investigate the impact of elevated c-di-AMP levels on host immune signaling, we examined the activation of the STING pathway and downstream cytokine responses in macrophages infected with MRSA *versus* MSSA strains. Fig. 4A outlines the experimental set up. Our results demonstrate that MRSA infection led to significant activation of the STING pathway, as evidenced by increased phosphorylation of STING and upregulation of Interferon-Stimulated gene 15 (ISG15) protein levels (Fig. 4B). Concomitantly, MRSA-infected macrophages exhibited markedly higher expression of Type I interferon (IFN-β) both at the transcript (Fig. 4C) and protein levels (Fig. 4D), compared to those infected with MSSA. In contrast, the expression of the pro-inflammatory cytokine Interleukin (IL)-1β was significantly suppressed in MRSA-infected macrophages, as shown by qRT-PCR (Fig. 4E). These findings suggest that heightened c-di-AMP production by MRSA activates the host STING signaling axis, skewing the innate immune response towards a Type I IFN-dominant profile while suppressing IL-1β-mediated inflammation. This immunomodulatory shift may favour intracellular survival of MRSA by dampening pro-inflammatory defences.

### 3.5 *In silico* molecular docking identifies FDA-approved drug candidates targeting DacA

**Fig. 5:**
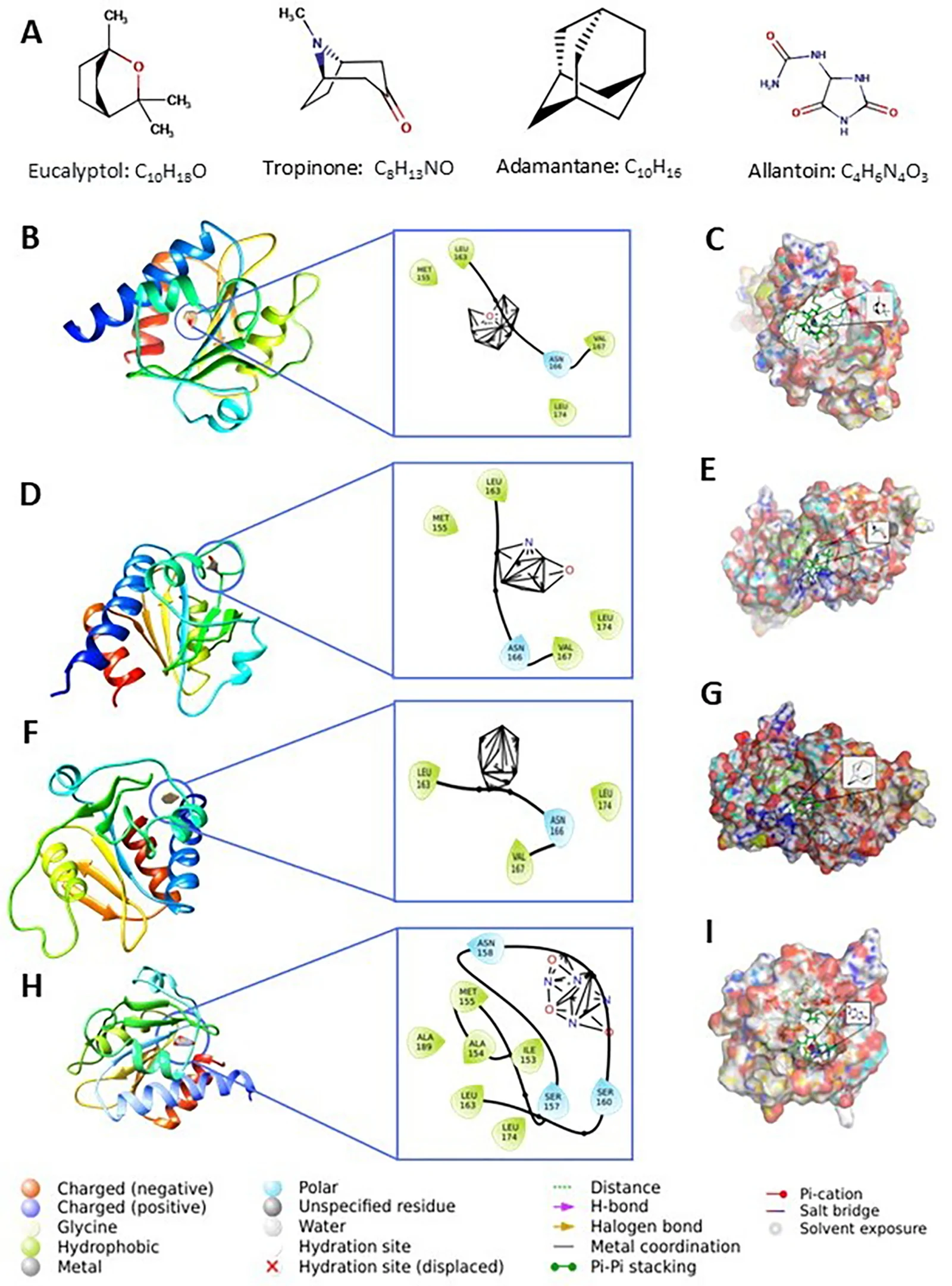
*In Silico* Molecular Docking of FDA-Approved Compounds with DacA from *S. aureus*. Molecular docking was performed using the crystal structure of DacA (PDB ID: 6GYW; 1.70 Å resolution) to identify potential inhibitors targeting the c-di-AMP cyclase active site. The figure depicts (A) the chemical formulas and structures of the four selected inhibitors: eucalyptol, tropinone, adamantane, and allantoin. (B, D, F, H) Ligand interaction, and (C, E, G, I) surface representation diagrams for eucalyptol, tropinone, adamantane, and allantoin with DacA, respectively, were generated using Schrödinger Maestro software.

To explore the potential of targeting c-di-AMP synthesis as a strategy to reduce biofilm formation and restore β-lactam susceptibility in MRSA, we performed in silico screening of FDA-approved bioactive and drug-associated small molecules for their ability to inhibit DacA. Molecular docking was carried out using the crystal structure of S. aureus DacA (PDB ID: 6GYW; resolution 1.70 Å). A virtual library of 10,614 FDA-approved compounds was screened, and the top 50 ligands were ranked based on their binding energy (BE) (Supplementary Data File 1). Further to ensure the appropriateness of the docking model, the ROC curve analysis was performed, which yielded an AUC value of 0.98, indicating that the model achieved perfect discrimination between active and inactive molecules (Supplementary Figure 3). Among the top 50 ligands, four compounds (Commercially accessible)- eucalyptol, tropinone, adamantane, and allantoin- were shortlisted for detailed interaction analysis. Fig. 5A depicts the chemical formula and structure of the four selected potential inhibitors. Among them, eucalyptol displayed the strongest binding affinity (–5.11 kcal/mol), forming hydrophobic interactions with Asn166 and Val167 within the cyclase domain and fitting tightly into a hydrophobic groove that could block substrate access (Fig. 5B, C). Tropinone, with a moderate binding energy of -4.02 kcal/mol, established interactions with Leu163, Asn166, and Val167, indicating its potential to interfere with catalytic function despite relatively lower affinity than eucalyptol or adamantane (Fig. 5D, E). Adamantane also demonstrated a favorable binding energy (-4.69 kcal/mol), engaging Leu163, Asn166, and Val167; its rigid cage-like structure allowed stable anchoring within the catalytic cleft, suggesting steric hindrance of enzymatic activity (Fig. 5F, G). In contrast, allantoin exhibited the lowest binding affinity (–3.92 kcal/mol) and localized within the cyclase domain through interactions with Ile153, Ala154, and Met155, stabilizing its position through polar and hydrogen-bond contacts (Fig. 5H, I). Collectively, these results highlight that all four compounds engage catalytically important residues within the cyclase domain of DacA as promising scaffolds for repurposing as inhibitors of c-di-AMP synthesis in *S. aureus*.

### 3.6 *In silico* analysis of pharmacokinetic and toxicological properties (ADMET analysis)

The *in silico* ADMET predictions indicate that all four compounds possess varied degrees of drug-like properties (Supplementary Data File 2). The physicochemical characteristics of the selected compounds were evaluated based on key molecular descriptors, including molecular weight (Daltons), number of hydrogen bond donors and acceptors, lipophilicity (Log P), aqueous solubility (S), surface area and the number of rotatable bonds. All compounds exhibited molecular weights below 500 Da, with rotatable bonds ranging from 0 to 3, well within the acceptable limit of ≤10. The number of hydrogen bond acceptors ranged from 0 to 3 (≤10), and hydrogen bond donors were between 0 and 3, staying below the threshold of 5. The Log P values ranged from −2.1798 to 2.8326, which are all within the desirable range of ≤5. Surface area values varied from 61.302 Å² to 66.741 Å², indicating favorable polarity. Collectively, these findings confirm that all compounds comply with Lipinski's Rule of Five, suggesting good oral bioavailability potential.

All four drugs were predicted to be non-inhibitors of P-glycoprotein I and II. The water solubility data indicated that all compounds are water-soluble. Caco-2 permeability was found to be high (>0.90) for eucalyptol (1.491), tropinone (1.351), and adamantane (1.364), while allantoin showed low permeability (<0.90) with a value of −0.271. In terms of human intestinal absorption, all four drugs showed high absorption percentages, with eucalyptol (100%), tropinone (98.793%), adamantane (94.151%), and allantoin (51.948%), all exceeding the 30% threshold. Eucalyptol (−2.709), tropinone (−2.924), and allantoin (−3.219) showed low skin permeability (LogKp < −2.5), whereas adamantane (−1.975) exhibited higher skin permeability (LogKp > −2.5). Eucalyptol and tropinone were identified as P-glycoprotein substrates, while adamantane and allantoin were predicted to be non-substrates.

The distribution of the selected drug molecules was evaluated using parameters such as volume of distribution in humans (VDss), fraction unbound, BBB permeability, and CNS permeability. Based on the VDss values, eucalyptol and adamantane were predicted to be distributed more in tissues, while tropinone and allantoin appeared to remain mostly in the plasma. All four compounds showed fraction unbound values that fall within acceptable limits, indicating a favourable binding profile with plasma proteins.

In the metabolism profile, none of the four compounds were predicted to act as substrates for CYP2D6 or CYP3A4, nor did they exhibit inhibitory activity against major cytochrome P450 enzymes, including CYP1A2, CYP2C19, CYP2C9, CYP2D6, and CYP3A4. These enzymes play a central role in hepatic drug metabolism, and the absence of such interactions suggests a lower likelihood of CYP-mediated metabolic interference. The excretion potential of the selected compounds was assessed based on total clearance and renal OCT2 substrate predictions. None of the compounds were identified as substrates for the renal OCT2 transporter, suggesting that their elimination is not facilitated by OCT2-mediated active transport. The predicted total clearance values varied among the compounds, with adamantane showing the lowest clearance (−0.004 log mL/min/kg), indicating poor elimination, and tropinone exhibiting the highest clearance (1.161 log mL/min/kg), reflecting efficient systemic removal.

All compounds were found to be AMES negative, indicating a non-mutagenic profile. The maximum tolerated dose (human) ranged from 0.048 for adamantane to 1.575 for allantoin. All the compounds were predicted to be non-inhibitors of hERG I and hERG II, suggesting a low risk of cardiotoxicity. Oral rat acute toxicity (LD50) and chronic toxicity (LOAEL) values were evaluated, with tropinone exhibiting potential chronic toxicity, having a LOAEL value of 0.531. None of the compounds were predicted to exhibit hepatotoxicity. Minnow toxicity results showed that all four compounds are non-toxic, with values greater than −0.3 log mM.

In addition, as shown in Supplementary Figure 4 A-D Radar plot analysis indicated that eucalyptol largely fell within the optimal range for oral bioavailability, displaying favorable lipophilicity, solubility, and molecular size, while tropinone satisfied most parameters but showed slight deviation in polarity. Adamantane displayed good oral bioavailability profile with minor concerns around high lipophilicity *vs* polarity balance. Allantoin, however, deviated in multiple dimensions, particularly size and polarity, reflecting limitations in its pharmacokinetic suitability. Further, BOILED-Egg prediction localized eucalyptol and adamantane in the yolk region, suggesting good intestinal absorption along with the ability to cross the BBB; tropinone fell in the white region, consistent with intestinal absorption but lacking BBB penetration; and allantoin appeared outside the egg, indicative of poor absorption (Supplementary Figure 4 E). In this analysis, all four compounds were predicted as PGP- substrates, suggesting they are not prone to efflux. These compounds have not previously been reported as DacA inhibitors and were thus selected for subsequent minimum inhibitory concentration (MIC) assessment against S. aureus.

### 3.7 Restriction of MRSA growth by the shortlisted compounds alone and in combination with methicillin

**Fig. 6:**
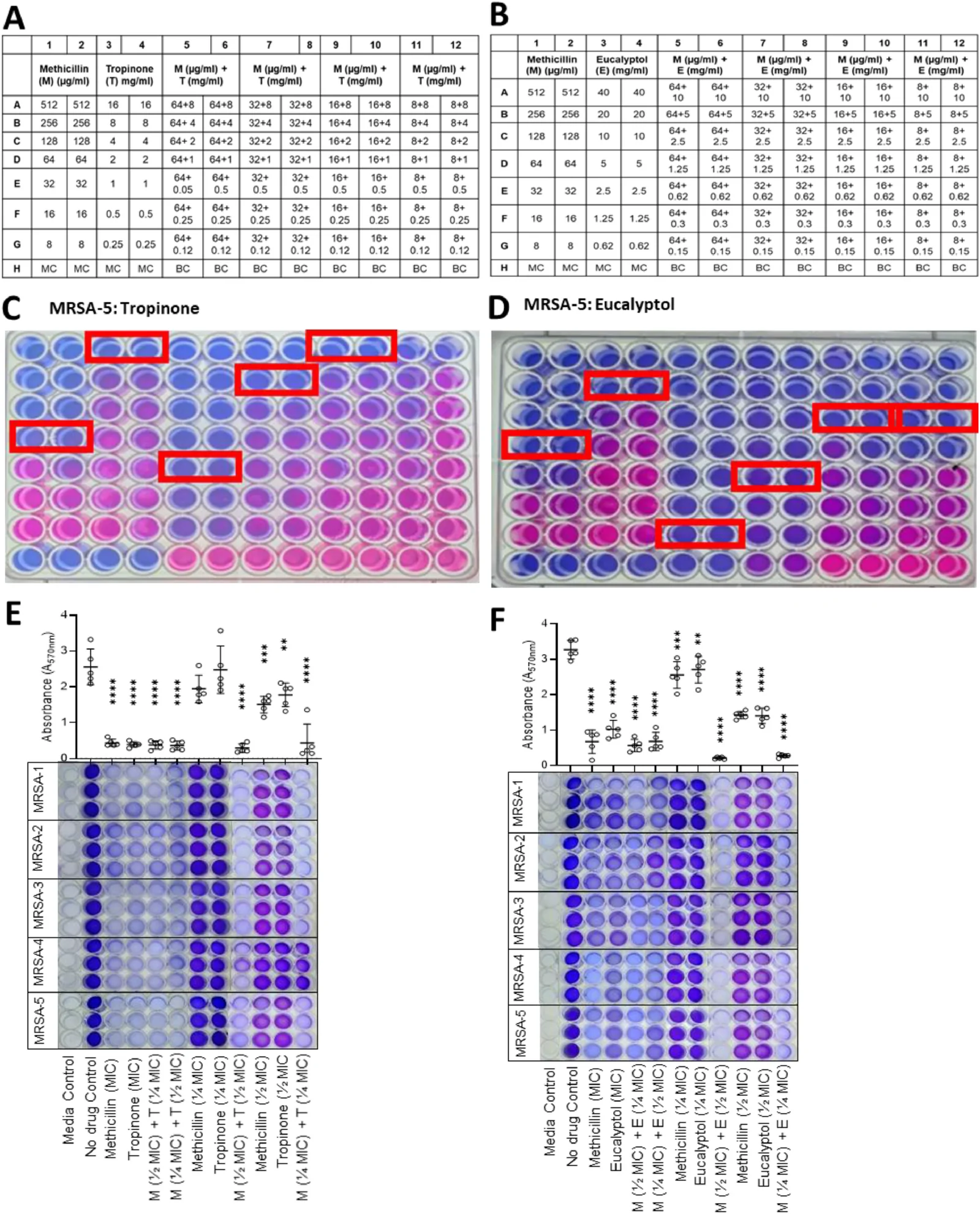
Antibacterial and Anti-Biofilm Activities of Tropinone and Eucalyptol Alone and in Combination with Methicillin Against MRSA. (A) and (B) depicts the outlines of the plate design for MABA assay with tropinone and eucalyptol, respectively. MC: Media control, and BC: Bacteria control. (C) and (D) depicts a representative MABA assay plate image of one of the MRSA isolates (MRSA-5) for tropinone and eucalyptol, respectively. (E & F) Crystal violet-based biofilm quantification assays were performed to evaluate the inhibitory effects of tropinone and eucalyptol on biofilm formation, alone or in combination with methicillin, under MIC and various sub-MIC conditions. Data are represented as scattered plot with mean ± SD, *n* = 5 biological replicates, each with technical triplicates. ****p < 0.0001, ***p < 0.001, **p < 0.01, *p < 0*.05*, One-way ANOVA with Dunnett's post hoc test for multiple comparisons. Media control data is not presented in the plot.

To evaluate the antibacterial potential of the shortlisted compounds identified through *in silico* docking, MIC assays were performed using the MABA assay alone or in combination with methicillin as described in the materials and methods section. Of the four candidate compounds, MIC values could be determined for tropinone and eucalyptol against five clinical MRSA strains. Due to solubility limitations of the commercially available compounds, MIC determination for adamantane and allantoin could not be performed under assay conditions. Fig. 6A and B depicts the outlines of the plate design for tropinone and eucalyptol, respectively. Fig. 6C and D depicts representative MABA assay plate image of one of the MRSA isolates (MRSA-5) for tropinone and eucalyptol, respectively, while for MABA plate images for other isolates (MRSA1–4) were provided as Supplementary Figure 4. Table-1 summarizes the overall MIC data. As standalone agents, tropinone and eucalyptol exhibited moderate inhibitory activity, with MIC values of 16 mg/ml (115 mM) and 20 mg/ml (129 mM), respectively. Notably, when tested in combination with methicillin, both compounds substantially reduced the MIC of methicillin across all tested MRSA strains (Table 1, Fig. 6C and D, and Supplementary Figure 4).

**Table 1:** Minimum Inhibitory Concentrations (MIC) of Tropinone and Eucalyptol Alone or in Combination with Methicillin Against MRSA Strains.

MRSA Strain	Methicillin (MIC) (µg/ml)	Tropinone (MIC) (mg/ml)	Eucalyptol (MIC) (mg/ml)	Methicillin (µg/ml) + Tropinone (mg/ml)	Methicillin (µg/ml) + Eucalyptol (mg/ml)
MRSA-1	128	16	20	64 (½ MIC) + 2 (⅛ MIC)	64 (½ MIC) + 0.15 (1/134 MIC)
				32 (¼ MIC) + 8 (½ MIC)	32 (¼ MIC) + 5 (¼ MIC)
MRSA-2	128	16	20	64 (½ MIC) + 4 (¼ MIC)	64 (½ MIC) + 0.3 (1/67 MIC)
				32 (¼ MIC) + 8 (½ MIC)	32 (¼ MIC) + 5 (¼ MIC)
MRSA-3	128	16	20	64 (½ MIC) + 2 (⅛ MIC)	64 (½ MIC) + 1.25 (1/16 MIC)
				32 (¼ MIC) + 8 (½ MIC)	32 (¼ MIC) + 5 (¼ MIC)
MRSA-4	128	16	20	64 (½ MIC) + 2 (⅛ MIC)	64 (½ MIC) + 2.5 (⅛ MIC)
				32 (¼ MIC) + 8 (½ MIC)	32 (¼ MIC) + 10 (½ MIC)
MRSA-5	64–128	16	20	64 (½ MIC) + 0.5 (1/32 MIC)	64 (½ MIC) + 0.15 (1/134 MIC)
				32 (¼ MIC) + 4 (¼ MIC)	32 (¼ MIC) + 0.625 (1/32 MIC)
				16 (⅛ MIC) + 8 (½ MIC)	16 (⅛ MIC) + 2.5 (⅛ MIC)

For example, in strain MRSA1–4, the methicillin MIC was reduced from 128 µg/ml to 32 µg/ml in the presence of sub-MIC concentration (½ MIC) of tropinone-corresponding to a 4-fold reduction (Table 1). Similarly, in MRSA5, methicillin MIC dropped from 64–128 µg/ml to 16 µg/ml when combined with sub-MIC concentration (½ MIC) of tropinone, also representing a 4–8-fold enhancement in susceptibility to methicillin. The combinatorial effect of eucalyptol showed a similar trend across four MRSA isolates (MRSA1 to 4). Notably, in isolate MRSA5, the methicillin MIC decreased from 64 to 128 µg/ml to 16 µg/ml when combined with as little as ⅛ of MIC concentration of eucalyptol, representing a 4- to 8-fold enhancement in susceptibility to methicillin. These results suggest a synergistic effect between DacA inhibitors and methicillin, likely due to impaired c-di-AMP signaling, leading to sensitization of MRSA strains to β-lactam antibiotics. These findings validate the therapeutic potential of targeting c-di-AMP biosynthesis to restore methicillin efficacy against resistant strains.

### 3.8 Prevention of MRSA biofilm formation by the shortlisted compounds alone and in combination with of methicillin

To investigate the impact of the shortlisted compounds on MRSA biofilm formation, we performed a high-throughput biofilm assay using both MIC, and sub-MIC concentrations of tropinone, eucalyptol, and methicillin, as well as in various combinations (Fig. 6E-F). Treatment with either the test compounds or methicillin at their respective MICs significantly inhibited biofilm formation (Fig. 6E-F).

Notably, a marked combinatorial effect was observed when sub-MIC concentrations of methicillin and the test compounds were used in combination. For instance, a combination of methicillin at either ½ MIC or ¼ MIC with tropinone (Fig. 6E) or eucalyptol (Fig. 6F) at ½ MIC or ¼ MIC resulted in strong inhibition of biofilm formation, comparable to that seen with full MIC levels of methicillin alone. These results indicate a synergistic interaction between methicillin and the DacA inhibitors, supporting the potential of these compounds to enhance the efficacy of existing antibiotics against biofilm-forming MRSA strains.

### 3.9 DacA binding and inhibition by tropinone and eucalyptol leading to reduced c-di-AMP levels in MRSA broth culture and in infected THP1 macrophages

**Fig. 7:**
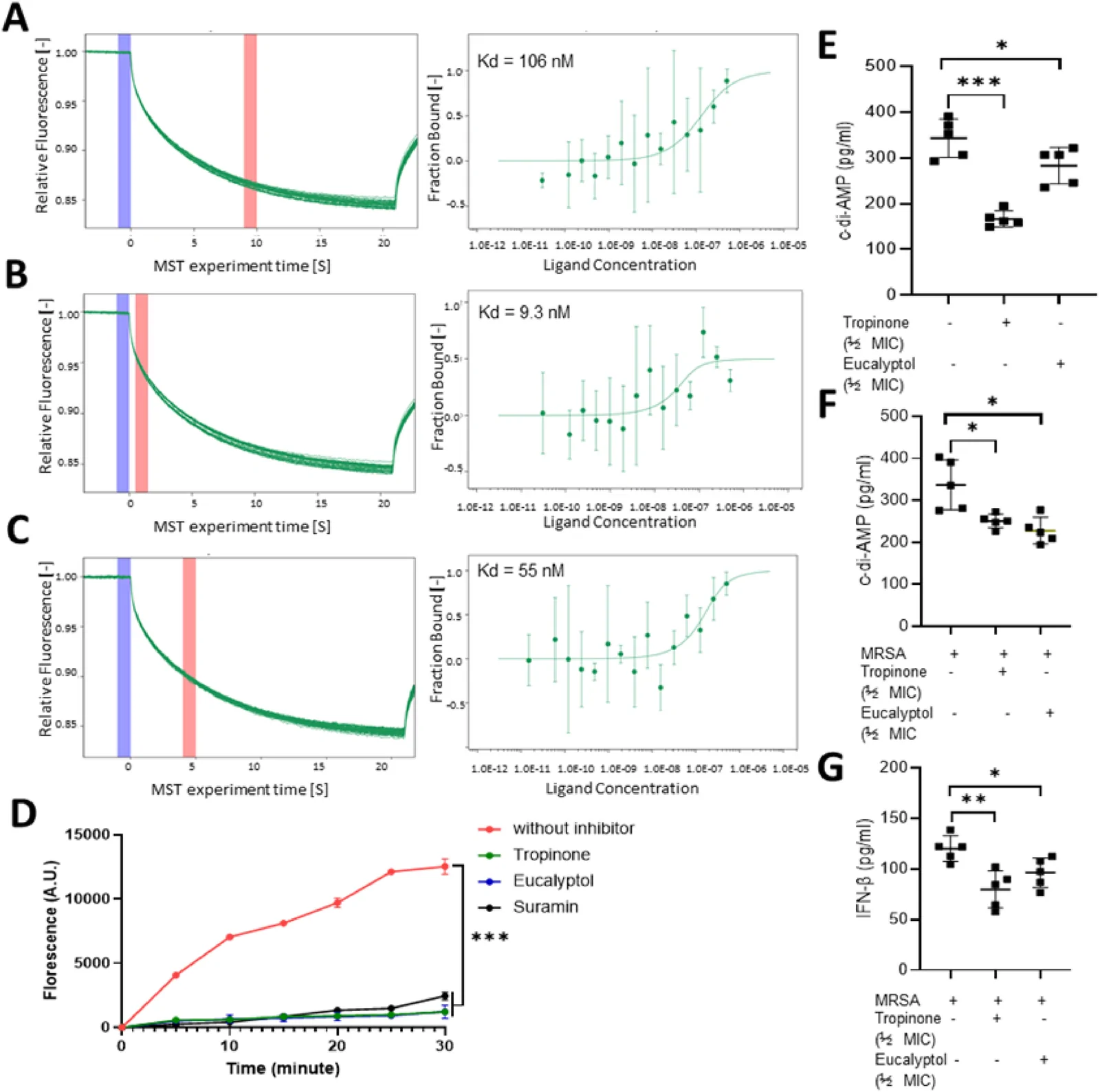
Assessing DacA Inhibition via biochemical assays, and Its Impact on c-di-AMP and IFN-β in MRSA and Host Cells. (A–C) Microscale thermophoresis (MST) traces corresponding to various concentrations and MST binding curves are shown for (A) tropinone, (B) Eucalyptol, and (C) Suramin, respectively. Regression analysis of normalized fluorescence data yielded dissociation constants (K_d_) for the inhibitors. The ligand binding data represent the mean ± SD from three independent experiments (*n* = 3). (D) Coralyne assay was used to evaluate the inhibitory activity of tropinone, eucalyptol, and suramin against DacA enzymatic function. Fluorescence intensity measured at λ ex = 420 nm and λ em = 475 nm in the presence of tropinone, eucalyptol, and suramin (20 µM each) and 1 µM DacA. Area under the curve (AUC) analysis was performed on the fluorescence kinetics, showing significant inhibition of enzymatic activity in the presence of all three inhibitors- Tropinone, Eucalyptol, and Suramin compared with the no-inhibitor control. Statistical significance was determined by one-way ANOVA followed by pairwise comparison against control using Welch’s *t*-test (*n* = 3, ***p < 0.001). (E) ELISA-based quantification of intracellular c-di-AMP in MRSA cultures treated with sub-inhibitory concentrations of tropinone and eucalyptol compared to untreated controls. (F) c-di-AMP levels in MRSA-infected THP-1 macrophages treated with sub-inhibitory concentrations of tropinone and eucalyptol compared to untreated controls. (G) IFN-β levels in supernatants of MRSA-infected THP-1 macrophages treated with sub-inhibitory concentrations of tropinone and eucalyptol compared to untreated controls. Data are presented as scatter plots showing individual biological replicates with mean ± SD, *n* = 5 biological replicates, each with technical duplicates. ***p < 0.001, **p < 0.01, *p < 0*.05*; Unpaired *t*-test (2-tailed).

To validate the direct interaction of these compounds with the target enzyme, we assessed the binding affinity of tropinone and eucalyptol to purified recombinant DacA using microscale thermophoresis (MST) using a NanoTemper equipment. The dissociation constant (Kd) was determined under optimized assay conditions as described in Materials and Methods. MST analysis revealed that tropinone binds to DacA with a Kd of 106 nM (Fig. 7A), while eucalyptol displayed significantly stronger binding with a Kd of 9.3 nM (Fig. 7B). As a reference, we included suramin, a previously reported DacA inhibitor (Opoku-Temeng and Sintim, 2016), which exhibited a Kd of 55 nM under the same conditions (Fig. 7C). These results demonstrate that both eucalyptol and tropinone possess high binding affinity for DacA, with eucalyptol showing superior interaction.

Further, to investigate whether these compounds inhibit DacA enzymatic activity, we employed a coralyne fluorescence-based assay, as previously described. This assay monitors the conversion of ATP to cyclic di-AMP (c-di-AMP) by DacA, and binding of c-di-AMP to coralyne leads to fluorescence signal at 420 nm (Ex)/475 nm (Em). In the presence of an inhibitor, c-di-AMP synthesis is blocked, resulting in reduced fluorescence. DacA activity was evaluated in the presence of tropinone and eucalyptol at a final concentration of 20 mM. Both compounds showed a significant inhibitory effect on DacA (1 μM), reducing fluorescence intensity by approximately 90% and 95%, respectively after 30 min, comparable to the known DacA inhibitor suramin, used here as a positive control (Fig. 7D) (Opoku-Temeng and Sintim, 2016). These findings provide strong biochemical evidence supporting DacA as the molecular target of these compounds and reinforce their therapeutic potential for repurposing against MRSA.

In addition, to assess the biological impact of the shortlisted DacA inhibitors on *S. aureus* during active growth, we measured c-di-AMP levels in MRSA cultures exposed to sub-inhibitory concentration (½ MIC) of tropinone and eucalyptol. As the MIC levels of the inhibitor completely inhibited bacterial growth, lower concentrations were used to evaluate partial inhibition of DacA activity. ELISA-based quantification revealed a significant reduction in c-di-AMP levels in the treated MRSA cultures compared to untreated controls (Fig. 7E), confirming effective inhibition of cyclic-di-AMP synthesis even at sub-MIC doses.

To further investigate the functional impact of DacA inhibition during infection, human THP1 macrophages were infected with the clinical MRSA isolates in the presence or absence of tropinone (½ MIC). After 6 h of infection, intra-macrophage c-di-AMP was quantified along with host IFN-β levels to assess downstream immune activation. Treatment with both tropinone and eucalyptol led to a notable decrease in intracellular c-di-AMP production (Fig. 7F), which was accompanied by a considerable reduction in IFN-β secretion from THP1 cells (Fig. 7G). These results confirm that DacA inhibition in MRSA by both the compounds remains effective during infection and alters host immune signaling by modulating type I interferon responses as well.

### 3.10 Safety and selectivity of lead compounds

**Fig. 8:**
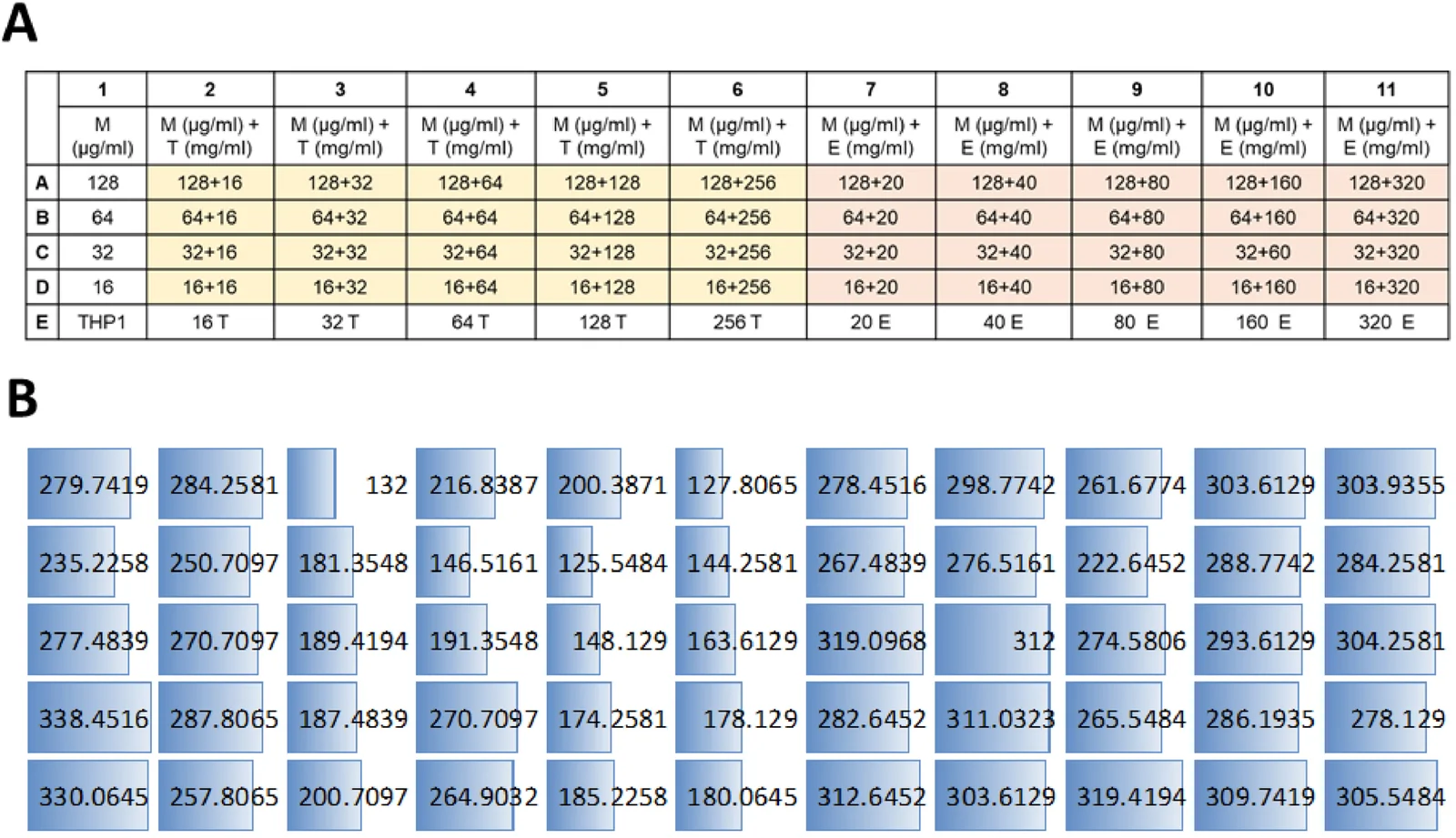
Evaluation of cytotoxicity of selected drugs by LDH release assay in THP-1 cells. Cytotoxicity of tropinone and eucalyptol alone or in combination with methicillin was assessed by the checkerboard method by measuring LDH release after 48 h of treatment. (A) Plate design for LDH release assay, serial 2-fold dilution of methicillin (128 to 16 µg/ml), tropinone (256 to 16 mg/ml), and eucalyptol (320 to 20 mg/ml) were used alone or in combination. (B) Heat map horizontal bar graph depiction of LDH levels (pg/ml) in each well following drug treatment individually or in combination with methicillin. The heat map is generated by using Microsoft Excel. (M = Methicillin, T = Tropinone, and E = Eucalyptol).

To assess the safety profile of the shortlisted DacA inhibitors, cytotoxicity assays were conducted in THP-1 macrophages using the LDH release assay. Both tropinone and eucalyptol were tested individually as well as in combination with methicillin in a checkerboard format (Fig. 8A). As shown in Fig. 8B, neither of the compounds exhibited any cytotoxic effects [depicted as LDH levels (pg/ml) comparable to that of un-treated THP1 cells] at their MIC values (for tropinone, 16 mg/ml, and for eucalyptol, 20 mg/ml) or up to 16-fold higher concentrations, indicating a broad therapeutic margin reflecting the inherently safe profile of these compounds within the tested concentration range. Importantly, co-treatment with methicillin did not increase cytotoxicity even at the highest concentration tested for both the compounds (for tropinone, 256 mg/ml, and for eucalyptol, 320 mg/ml), further supporting the safety of their combined use. Further, selectivity index (SI) analysis demonstrated favorable SI estimates for both tropinone and eucalyptol either alone or in combination with methicillin, supporting their potential utility as safe adjunctive anti-MRSA therapeutic agents (Supplementary Table 4). Collectively, these findings suggest that tropinone and eucalyptol are well tolerated by mammalian cells, selective in their activity against bacterial targets, and suitable candidates for advancement into further evaluation as potential anti-MRSA therapeutics.

## 4 Discussion

This study provides insights into the role of c-di-AMP signaling in the context of methicillin resistance and biofilm formation in *S. aureus*, particularly MRSA strains. Our findings support the hypothesis that elevated c-di-AMP levels not only associate with antimicrobial resistance but also facilitate immune evasion through modulation of host cytokine responses, highlighting c-di-AMP biosynthesis as a potential non-canonical therapeutic target.

A key observation in our study was the positive correlation between methicillin MICs and biofilm biomass across clinical MRSA isolates, suggesting that the c-di-AMP pathway may coordinate both phenotypic drug resistance and persistence traits. This aligns with prior studies that demonstrated the regulatory role of c-di-AMP in biofilm formation and cell wall homeostasis (Corrigan et al., 2013; Whiteley et al., 2017). Moreover, transcriptional upregulation of *cdaA* and other c-di-AMP signaling-associated genes under biofilm and antibiotic stress conditions indicates an active remodeling of bacterial regulatory networks to support survival, as previously observed in several other bacteria (Dengler Haunreiter et al., 2023; Dey et al., 2020; Kundra et al., 2021; Witte et al., 2013). It is noteworthy to mention here that regulation of c-di-AMP homeostasis in *S. aureus* is not governed solely at the transcriptional level but also involves post-transcriptional modulation of DacA activity through interacting proteins such as YbbR and GlmM (Tosi et al., 2019). Previous studies have demonstrated that GlmM directly interacts with DacA and inhibits its cyclase activity, thereby contributing to fine-tuning of intracellular c-di-AMP levels under stress conditions (Pathania et al., 2021). Similarly, YbbR has been implicated in the regulation of DacA activity in response to cell envelope-associated signals. In the present study, the simultaneous increase in intracellular c-di-AMP levels along with elevated *gdpP* expression under methicillin stress suggests the existence of a dynamic compensatory regulatory network aimed at maintaining c-di-AMP homeostasis. Therefore, the observed transcriptional upregulation of *cdaA* and related pathway genes alone may not fully explain the altered c-di-AMP levels, and additional post-transcriptional regulatory mechanisms likely contribute to the overall signaling dynamics in MRSA during antibiotic stress.

Importantly, the immunomodulatory role of c-di-AMP during infection was substantiated by our findings that MRSA-infected macrophages produced elevated levels of IFN-β and reduced levels of IL-1β, consistent with STING pathway activation. These results corroborate earlier reports demonstrating c-di-AMP-induced type I interferon production via STING sensing (Dey et al., 2015; Woodward et al., 2010), which can alter protective inflammatory responses and favor chronic infection (Tang et al., 2022).

Another key aspect of this study involved evaluating the impact of sublethal methicillin exposure on MRSA physiology, particularly in the context of c-di-AMP signaling and antimicrobial resistance. Clinically relevant sub-MIC antibiotic levels, often arising from poor drug penetration or improper dosing, are known to trigger adaptive stress responses that drive resistance and biofilm formation (Andersson and Hughes, 2014; Penesyan et al., 2021). In our study, sub-MIC methicillin markedly increased intracellular c-di-AMP and upregulated *cdaA* and *gdpP* expression in MRSA, indicating activation of its c-di-AMP regulatory network supporting cell wall homeostasis and persistence. Methicillin stress also enhanced biofilm formation, consistent with β-lactam-induced tolerance (Kaplan, 2011). These findings highlight c-di-AMP as a central node linking antibiotic stress, biofilm persistence, and host-pathogen interactions, underscoring its potential as a therapeutic target.

Our identification of eucalyptol (1,8-cineole) and tropinone as potent DacA inhibitors is particularly noteworthy. Eucalyptol is a monoterpenoid ether- commonly found in eucalyptus oil- with FDA approval as a flavoring agent and ingredient in mouthwashes and decongestants (Gopal, 2015). Tropinone, an 8-azabicyclo[3.2.1] octan-3-one alkaloid with a bicyclic tropane scaffold, is historically used as a synthetic precursor in tropane alkaloid production (Atropine, scopolamine) and features a stable reaction framework (Rodriguez et al., 2021). Neither has previously been reported as a DacA inhibitor. Additionally, while our study relied on *in silico* predictions for ADMET profiling including bioavailability, previous experimental studies have demonstrated that eucalyptol is systemically absorbed and exhibits measurable pharmacokinetic properties *in vivo*, including brain distribution following oral administration (Dao et al., 2023; Sa et al., 2021). However, similar experimental bioavailability data for tropinone remain limited, highlighting the need for future *in vitro* and *in vivo* validation studies.

The ATP-binding site (Asp176–Gly177–Ser178) of *S. aureus* DacA lies within its conserved DisA_N cyclase domain and includes the key “DGA” motif, which directly interacts with ATP phosphates and coordinates Mg²⁺ for catalysis (Tosi et al., 2019). Structural studies of the catalytic domain (PDB ID: 6GYW) show that, in the absence of ATP, the two monomers remain apart, but ATP binding drives a head-to-head dimerization essential for condensing two ATP molecules into c-di-AMP (Tosi et al., 2019). Blocking this site- either by natural regulators like GlmM (Pathania et al., 2021) or by small molecules- can prevent ATP access or disrupt dimerization. We show that both eucalyptol and tropinone interact with the critical DisA-N domain, with eucalyptol demonstrating a particularly strong binding affinity (Kd = 9.3 nM). Consequently, the coralyne fluorescence assay demonstrated reduced c-di-AMP synthesis in the presence of the inhibitors. These findings align with previous reports of DacA inhibitors such as suramin, which also bind the catalytic pocket (Opoku-Temeng and Sintim, 2016). While detailed crystallographic validation of the compound-bound DacA complex is still warranted, the current binding data and inhibition profile suggest that these compounds likely act through orthosteric inhibition by competing with ATP at the active site.

Although cyclic di-AMP is recognized as a key signaling molecule in Gram-positive pathogens, development of DacA inhibitors remains limited. Suramin was among the first reported inhibitors but showed poor specificity and pharmacokinetics (Opoku-Temeng and Sintim, 2016). Subsequent *in silico* and *in vitro* screens identified candidates with limited validation, lacking studies in clinical MRSA or infection models (Li et al., 2022). Unlike earlier enzyme-only assays, we confirmed target engagement through MST and coralyne fluorescence assays, and demonstrated suppression of c-di-AMP synthesis in cultures and infected macrophages. We show, DacA inhibition reduces methicillin resistance, biofilm formation, and STING-mediated immune activation, supporting its translational potential and positioning c-di-AMP modulation as a therapeutic strategy against MRSA. In addition, the phenotypic reversal of MRSA highlights the potential of DacA inhibitors as adjuncts to conventional β-lactam therapy, especially considering the poor efficacy of monotherapy in biofilm-associated MRSA infections (Andersson and Hughes, 2014).

Despite promising findings, this study has certain limitations. Tropinone and eucalyptol showed high MICs in monotherapy, indicating their role as potentiators rather than standalone antibiotics. Potency may improve through SAR optimization or combination with efflux pump inhibitors. *In vivo* efficacy and pharmacokinetic evaluation are still required to establish translational potential. Off-target effects also warrant caution: eucalyptol can modulate CYP450 enzymes and cause neurotoxicity at high doses (Sharma et al., 2024), while tropinone derivatives may exhibit anticholinergic effects on cardiovascular and neural systems (De Nijs et al., 2023; Wang et al., 2023). Although no mammalian cytotoxicity was observed, comprehensive profiling of hepatic metabolism, neurotoxicity, and anticholinergic activity is needed before clinical development.

## 5 Conclusion

This study elucidates the functional relevance of c-di-AMP signaling in methicillin resistance, biofilm formation, and host-pathogen interactions in MRSA. Elevated c-di-AMP levels under antibiotic and biofilm stress correlated with increased resistance and persistence, indicating its role in adaptive bacterial survival. Upregulation of *cdaA* and *gdpP* suggests dynamic regulation to maintain intracellular c-di-AMP balance. Functionally, c-di-AMP–mediated activation of the host STING pathway enhanced IFN-β and suppressed IL-1β, facilitating immune evasion. Structure-based screening of FDA-approved drugs identified tropinone and eucalyptol as novel diadenylate cyclase (DacA) inhibitors that lowered c-di-AMP levels, disrupted biofilms, and restored β-lactam susceptibility without cytotoxicity. These results validate DacA as a tractable drug target and support pharmacological modulation of bacterial nucleotide signaling as a promising adjunctive approach to counter MRSA and related Gram-positive pathogens.

## Ethics statement

All experiments were reviewed and approved by the Institutional Biological Safety Committee (IBSC) of the National Institute of Animal Biotechnology, Hyderabad (Approval No IBSC/2019/NIAB/BDey001).

## Software and database

All the databases and software information are provided within the relevant sections of the manuscript.

## Disclosure statement

No potential conflict of interest was reported by the author(s).

## Data availability statement

All relevant data were presented in the manuscript as Figures, Tables, Supplementary tables or as Supplementary data files. All other data supporting the findings of this study are available from the corresponding author upon reasonable request.

## Funding

This study received financial support from the Indian Council of Medical Research (ICMR), Govt. of India, Adhoc Extramural Project (AMR/ADHOC/187/2019-ECD-II).

## Declaration of generative AI and AI-assisted technologies in the writing process

During the preparation of this work the author(s) used ‘grammarly’ and ‘ChatGPT’ in order to improve the readability and language of the manuscript. After using this tool, the author(s) reviewed, and edited the content thoroughly as needed and take(s) full responsibility for the content of the published article.

## Abbreviations

MRSA, Methicillin-resistant Staphylococcus aureus; MSSA, Methicillin-sensitive Staphylococcus aureus; c-di-AMP, cyclic di-adenosine monophosphate; DacA, diadenylate cyclase; GdpP, cyclic di-AMP phosphodiesterase; STING, stimulator of interferon genes; MABA, microplate Alamar Blue assay; ADME, absorption, distribution, metabolism, and excretion; NFW, nuclease-free water; MST, microscale thermophoresis; SAR, structure–activity relationship; CFU, colony-forming unit.

## CRediT author statement

Niti Kumari: Conceptualization, Methodology, Investigation, Formal analysis, Data curation, Writing – original draft. Itishree Jali: Conceptualization, Methodology, Investigation, Formal analysis, Data curation, Writing – original draft. Priyanka Garg: Investigation, Formal analysis. Repally Ayanna: Investigation. Vinay Bhaskar: Investigation. Vasundhra Bhandari: Resources. Shailesh Sharma: Conceptualization, Methodology, Investigation, Formal analysis, Resources. Bappaditya Dey: Conceptualization, Methodology, Investigation, Formal analysis, Resources, Supervision, Project administration, Writing – review & editing, Funding acquisition.

## Declaration of competing interest

The authors declare the following financial interests/personal relationships which may be considered as potential competing interests: Bappaditya Dey reports financial support was provided by Indian Council of Medical Research. If there are other authors, they declare that they have no known competing financial interests or personal relationships that could have appeared to influence the work reported in this paper.

## Appendix Supplementary materials

Image, application 1

Image, application 2

Image, application 3
