# Supplementary material for: Targeting cyclic di-AMP signaling through diadenylate cyclase inhibition reduces methicillin resistance in clinical MRSA isolates
Source: Curr Res Microb Sci. 2026 May 25;11:100615. doi: 10.1016/j.crmicr.2026.100615 (PMC13266020; doi:10.1016/j.crmicr.2026.100615)
Supplement: Supplementary file 3 [file mmc3.pdf]

Supplementary Materials

Targeting c-di-AMP Signaling through Diadenylate Cyclase Inhibition Reduces Methicillin Resistance in Clinical MRSA Isolates

Niti Kumari[1,2], Itishree Jali[1], Priyanka Garg[1], Repally Ayanna[1,#], Vinay Bhaskar[1,$], Vasundhra Bhandari[1,§], Shailesh Sharma[1,2], Bappaditya Dey[1,2*]

[1]BRIC-National Institute of Animal Biotechnology, Hyderabad, Telangana, India

[2]Regional Centre for Biotechnology, Faridabad, Haryana, India

Current Address:

[#]Department of Chemical & Material Engineering, University of Alabama, Huntsville, Alabama, USA

[$]Faculty of Veterinary Medicine, University of Calgary, Calgary, Canada

[§]Department of Pharmacoinformatics, National Institute of Pharmaceutical Education and Research, Hyderabad, Telangana, India

***Corresponding author**
Bappaditya Dey
bdey@niab.org.in
ORCiD ID: 0000-0003-2728-4683

Supplementary Figure 1

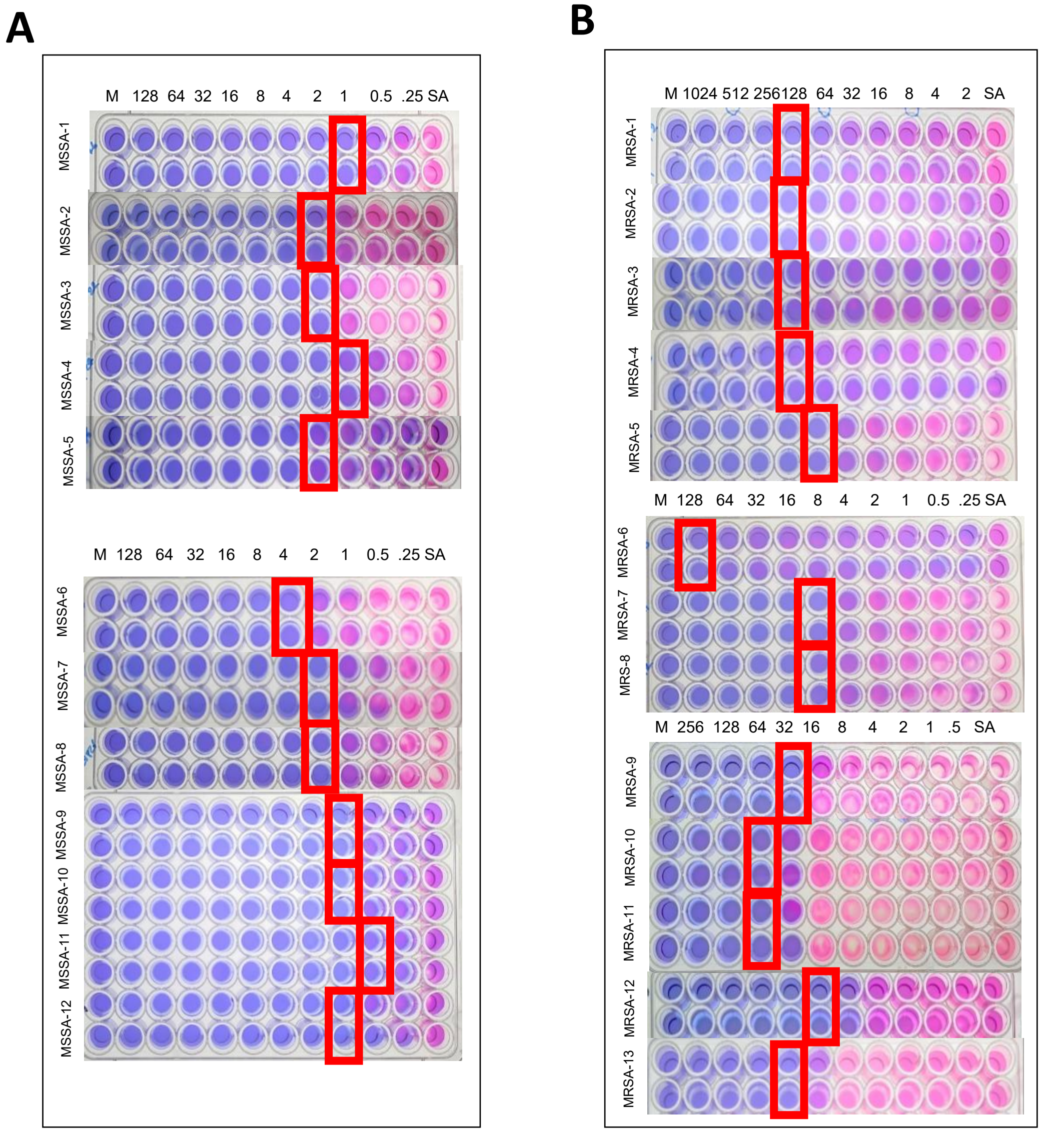

Supplementary Figure 1. Determination of MIC of Methicillin on *S. aureus* Clinical Isolates by MABA. Microplate Alamar Blue Assay (MABA) was performed to assess the antibacterial activity of methicillin against MSSA and MRSA *S. aureus* clinical isolates (n=25). Representative images show the colorimetric results of two-fold serial dilutions of methicillin, ranging from (A) 64–0.125 µg/mL for MSSA and 1024–2 and (B) 256-0.5 µg/mL for MRSA. MIC values ranged from 0.5–4 µg/mL for MSSA and 8–128 µg/mL for MRSA.

Supplementary Figure 2

A

	Methicillin resistance associated canonical genes (Present /absent)			
SA Strains	*mecA*	*fmtA*	*blaZ*	*femA*
MSSA1	Absent	Present	Present	Present
MSSA2	Present	Absent	Absent	Present
MSSA3	Absent	Absent	Present	Present
MSSA4	Absent	Present	Present	Present
MSSA5	Present	Present	Present	Present
MRSA1	Present	Present	Present	Present
MRSA2	Present	Present	Present	Present
MRSA3	Present	Present	Present	Present
MRSA4	Present	Present	Present	Present
MRSA5	Present	Present	Present	Present

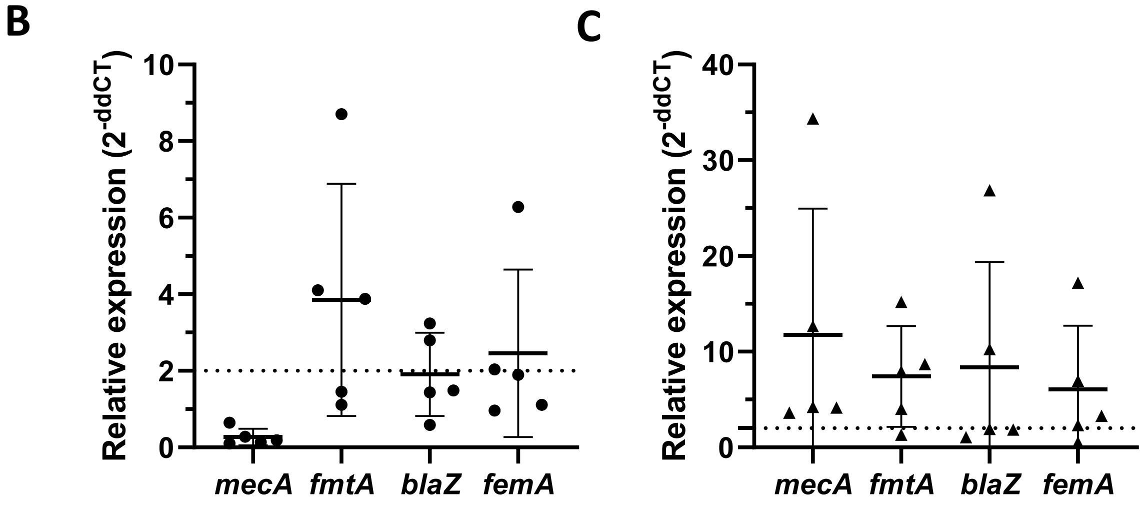

Supplementary Figure 2. Presence and Relative Gene Expression of Methicillin Resistance Associated Genes in Clinical *S. aureus* Isolates. (A) PCR-based detection of the presence or absence of the target genes; (B) Relative expression levels of methicillin resistance-associated genes (*mecA, fmtA, blaZ, and femA*) were analyzed in MRSA isolates compared to MSSA isolates in the logarithmic growth phase; and (C) Relative expression levels of these genes in MRSA when exposed to sub-MIC methicillin concentration compared to untreated bacteria. CT values from MSSA and MRSA isolates were first normalized to the housekeeping gene *rpoB*, and subsequently, relative gene expression fold changes were calculated using the 2^−ΔΔCt method. Data are presented as scatter plots showing individual biological replicates with mean ± SD (n = 5; each with technical duplicates).

Supplementary Figure 3

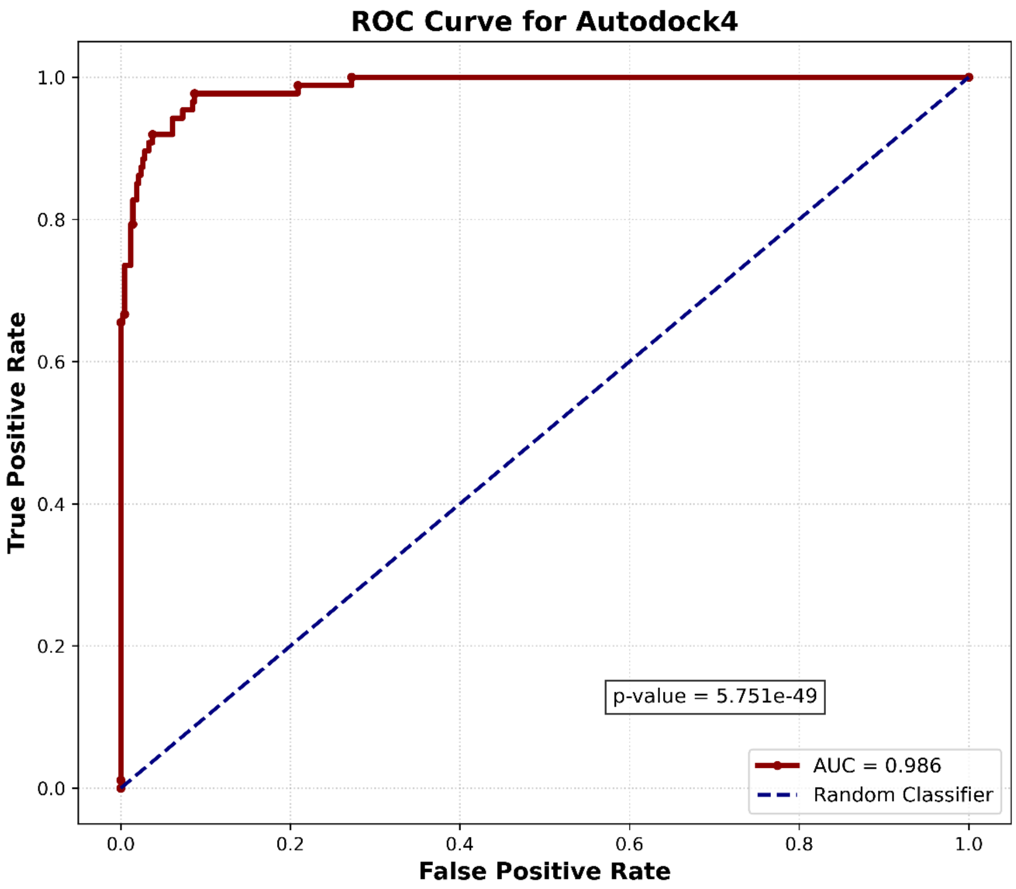

Supplementary Figure 3. Receiver operating characteristic (ROC) curve analysis evaluating the screening performance of the AutoDock4 docking model for DacA-targeted virtual screening. The top 50 ligands along with RDKit-generated decoy molecules were subjected to docking against DacA, and sensitivity and specificity were calculated based on classification of active and inactive compounds. The ROC analysis yielded an area under the curve (AUC) value of 0.986, indicating excellent discriminatory capability of the docking model in distinguishing active ligands from inactive molecules. The dashed diagonal line represents the performance of a random classifier.

Supplementary Figure 4

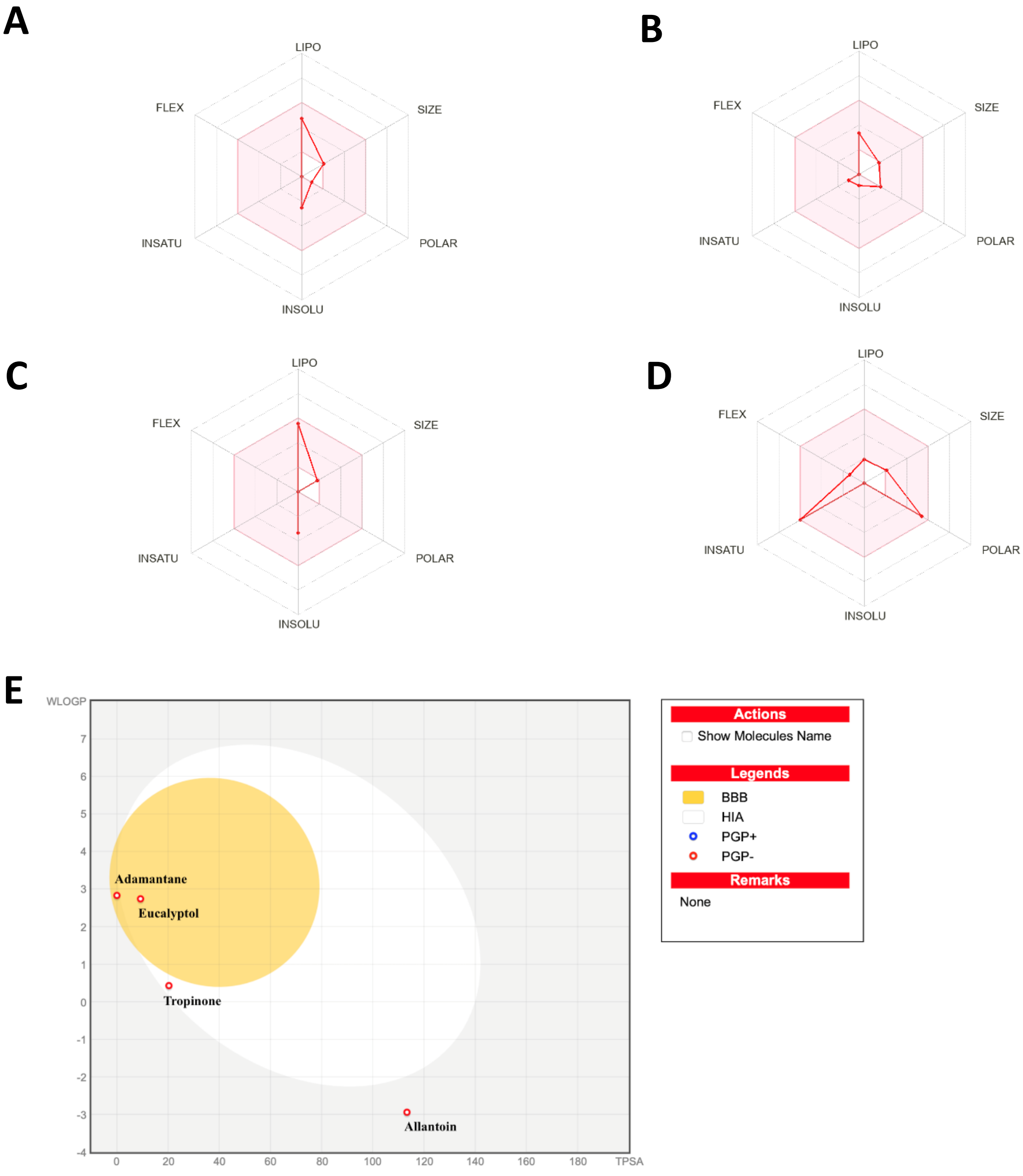

Supplementary Figure 4. Bioavailability radar plots, and BOILED-Egg prediction of shortlisted compounds. (A–D) Bioavailability radar plots of (A) Eucalyptol, (B) Tropinone, (C) Adamantane, and (D) Allantoin. The pink zone in the radar plot represents the optimal range for six physicochemical properties: lipophilicity (−0.7 ≤ XLOGP3 ≤ 5.0), size (150 ≤ MW ≤ 500 g/mol), polarity (20 ≤ TPSA ≤ 130 Å²), solubility (logS ≤ 6), saturation (fraction of sp^3 carbons ≥ 0.25), and flexibility (rotatable bonds ≤ 9). (E) BOILED-Egg prediction of Eucalyptol, Tropinone, Adamantane, and Allantoin. The plots were generated using SwissADME.

Supplementary Figure 5

A

B

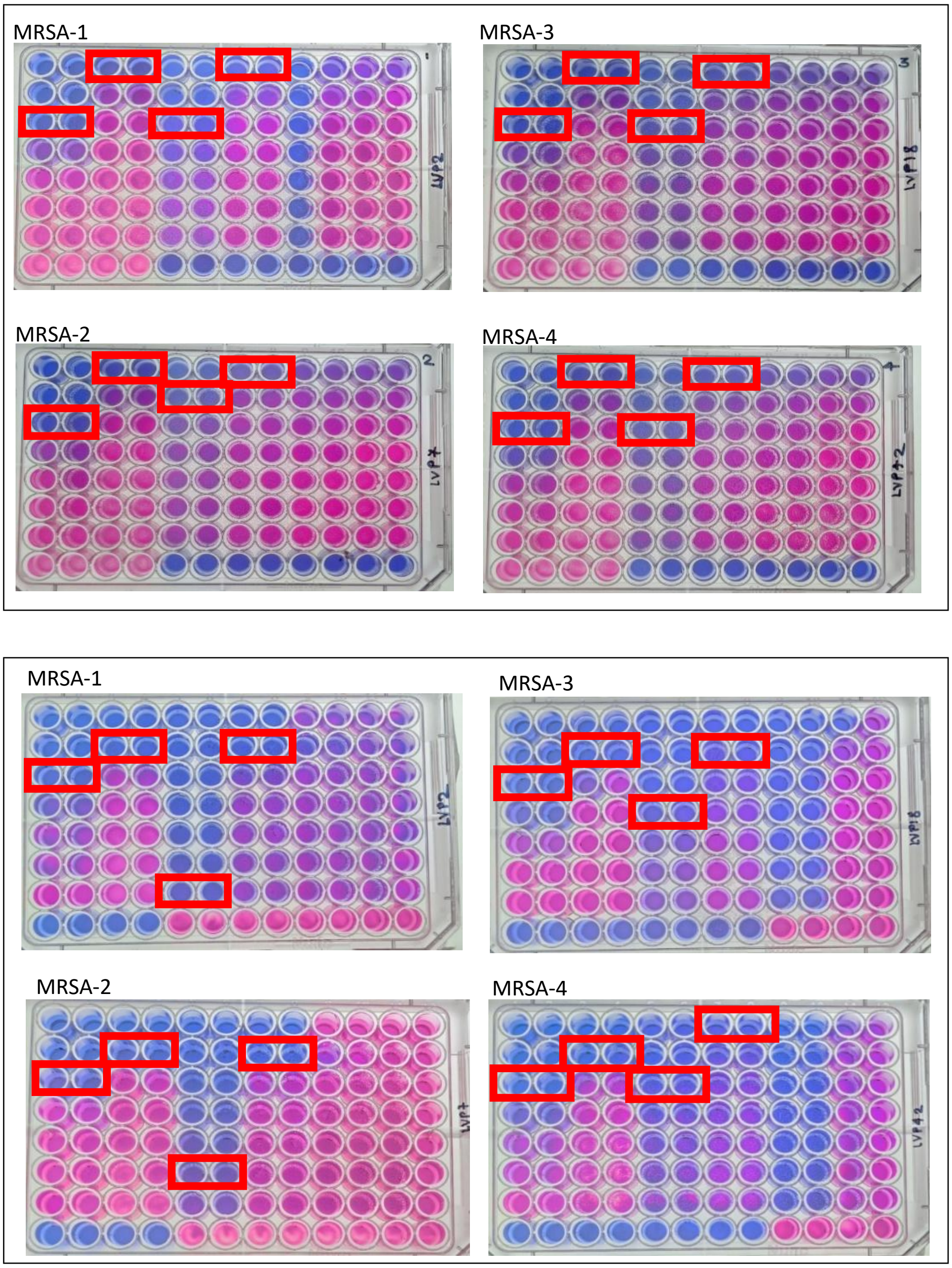

Supplementary Figure 5. Evaluation of Antibacterial Activity of Shortlisted Compounds in Combination with Methicillin Against *Staphylococcus aureus*. Representative Microplate Alamar Blue Assay (MABA) results showing the antibacterial activity of Tropinone and Eucalyptol in combination with methicillin against *MRSA* isolates no. 1-4. The assay was performed at least twice with two technical replicates in each case. Methicillin (512–8 µg/mL), Tropinone (16–0.25 mg/mL), and Eucalyptol (40–0.625 mg/mL) were tested either alone or in combination. (A) Methicillin (64–8 µg/mL) was tested in combination with serially diluted concentrations of Tropinone (8–0.125 mg/mL). (B) Methicillin (64–8 µg/mL) was tested in combination with serially diluted concentrations of Eucalyptol (10–0.15 mg/mL). A synergistic enhancement in methicillin activity was observed when combined with either Tropinone or Eucalyptol, as described in **Table 1** (main text).

Supplementary Table 1

Cloning primers			
S.no	Gene	Primer	Sequence
1	dacA	Forward primer	CGCGGATCCATATGGATTTTTCCAACTTTTTT
		Reverse primer	ATTAAGCTTTTTCACACCTTTCTTTTGAAAG
**Real-time PCR primers (Bacterial)**			
2	*rpoB*	Forward primer	GCGAACATGCAACGTCAAG
		Reverse primer	GACCTCTGTGCTTAGCTGTAATAAGC
3	*cdaA*	Forward primer	TTCAAGGCACGAAGATTGCAG
		Reverse primer	CCAACCGCAGCTCTATGTCT
4	*gdpP*	Forward primer	GTTGTTGCGAGACGTGAAGA
		Reverse primer	TTAAATGTCCACCGCCACCA
5	*ktrA*	Forward primer	ATTGGGGCAGATACGGTTGT
		Reverse primer	TGCTCGTCTGCCAACTCAAG
6	*cpaA*	Forward primer	ACGACTAGGTCTGTTTGACCG
		Reverse primer	CGTCCGTTGTGCTTTCAAGT
7	*kimA*	Forward primer	GTACAGGCAACGACCGTGAT
		Reverse primer	GCCTAAGCGATCACCTCTAACA
8	*trk*	Forward primer	AATGTTGCGAGTGCAAGTGT
		Reverse primer	ATGATAGACTGCCCCGCCAT
9	*pstA*	Forward primer	CGCCACTTGTGCTAATCGGT
		Reverse primer	TTGAGGCATTTTCGCCCAAG
10	*darA*	Forward primer	GGCAACAACAGGTGGGTTTT
		Reverse primer	GCCTCCCATAGGTGTAATAGGTG
11	*nrdR*	Forward primer	GGACTTGTGCGTTCTTGTGA
		Reverse primer	TCGTGAAGACACTTCCGTATGA
12	*kdpD*	Forward primer	CGTATGCCCACCTAAGCGAA
		Reverse primer	TGCTCCCACTAATTGCCACA
13	*pycA*	Forward primer	CCACGCCACCTTAGAACTGA
		Reverse primer	CGAATACTGTCCACCAGGCA
14	*glmM*	Forward primer	TTGGTGCAGAAGTGATGCGA
		Reverse primer	GCAACTGGATTATGAGAGGCTG
15	*ybbR*	Forward primer	AGCGAAGTTGATGCAGAAGTAGA
		Reverse primer	GCCTTCGTTTCACTTGGTTGT
16	*mecA*	Forward primer	GGCAGACAAATTGGGTGGTT
		Reverse primer	ACCTGAGATTTTGGCATTGTAGC
17	*blaZ*	Forward primer	AGGGCCAATCTGAACCTATTGT
		Reverse primer	TACACTCTTGGCGGTTTCACT
18	*fmtA*	Forward primer	TCGATTACAGACGAAGACACATTC
		Reverse primer	CGGCGCAACCTTTTCCTTATT
19	*femA*	Forward primer	ACAGCTAAAGAGTTTGGTGCCT
		Reverse primer	GCAAGCTGCAATGACCTCGT
**Real-time PCR primers (Host)**			
20	*IFN-β*	Forward primer	CTTGGATTCCTACAAAGAAGCAGC
		Reverse primer	TCCTCCTTCTGGAATGCTGCA
21	*IL-1β*	Forward primer	CCACAGACCTTCCAGGAGAATG
		Reverse primer	GTGCAGTTCAGTGATCGTACAGG
22	*β-actin*	Forward primer	CACCATTGGCAATGAGCGTTC

Supplementary Table 2

**Supplementary Table 2: MIC & other details of *Staphylococcus aureus* clinical isolates**				
Isolate Code	Source of isolates	MIC (µg/ml)	Methicillin Phenotype	Study Code
LVP16	Human	1	Sensitive	MSSA-1
LVP17	Human	2-4	Sensitive	MSSA-2
LVP22	Human	2	Sensitive	MSSA-3
LVP24	Human	1	Sensitive	MSSA-4
LVP66	Human	2-4	Sensitive	MSSA-5
LVP2	Human	128	Resistant	MRSA-1
LVP7	Human	128	Resistant	MRSA-2
LVP18	Human	128	Resistant	MRSA-3
LVP42	Human	128	Resistant	MRSA-4
LVP85	Human	64-128	Resistant	MRSA-5
LVP8	Human	128	Resistant	MRSA-6
LVP11	Human	8	Resistant	MRSA-7
LVP12	Human	8	Resistant	MRSA-8
LVP69	Human	2-4	Sensitive	MSSA-6
LVP77	Human	2	Sensitive	MSSA-7
LVP26	Human	2	Sensitive	MSSA-8
KA1	Bovine	1	Sensitive	MSSA-9
KA6	Bovine	1	Sensitive	MSSA-10
KA8	Bovine	0.5	Sensitive	MSSA-11
KA31	Bovine	1	Sensitive	MSSA-12
SA23	Bovine	32	Resistant	MRSA-9
SA72	Bovine	64	Resistant	MRSA-10
SA77	Bovine	64	Resistant	MRSA-11
SA79	Bovine	16	Resistant	MRSA-12
SA71	Bovine	32	Resistant	MRSA-13

Supplementary Table 2: Clinical Isolates of *Staphylococcus aureus*, Source, Minimum Inhibitory Concentration (MIC), Methicillin Phenotype, and assigned Study Codes.

Supplementary Table 3

**Supplementary Table 3: Cyclic-di-AMP Signaling Pathway Associated Genes, their Functions, and Supporting References.**		
**Name**	**key features**	**References**
cdaA	Diadenylate cyclase, synthesizes cyclic di-AMP and is involved in cell wall homeostasis and stress response	[33]
gdpP	Phosphodiesterase that regulates intracellular c-di-AMP levels, influencing β-lactam antibiotic tolerance	[34]
ktrA	Potassium transporter regulator that binds c-di-AMP, leading to conformational changes that inhibit K^+ uptake	[35]
cpaA	Cation–proton antiporter whose RCK_C domain binds cyclic-di-AMP to modulate ion (K^+/H^+) exchange activity	[36]
kimA	Potassium transporter whose activity is inhibited when c-di-AMP binds, potassium homeostasis	[37]
trk	Regulatory subunit of a potassium transporter and maintain potassium homeostasis.	[10]
pstA	c-di-AMP receptor protein with a ferredoxin-like fold that binds c-di-AMP and undergoes structural changes, and is associated with nitrogen metabolism.	[38]
darA	PII-like signal transducer that functions as a high-affinity receptor for c-di-AMP	[44]
nrdR	Repressor of the ribonucleotide reductase operon (nrdIEF), and it indirectly promotes c-di-AMP production by repressing NrdEF, which negatively regulates c-di-AMP levels	[39]
kdpD	Membrane-bound histidine kinase that binds c-di-AMP and inhibits the activation of the kdpFABC operon, thereby acting as a negative regulator of potassium uptake under salt stress conditions.	[40]
pycA	c-di-AMP–binding enzyme that links central carbon metabolism to nucleotide signaling by modulating pyruvate and oxaloacetate levels, thereby affecting amino acid biosynthesis and metabolic homeostasis.	[41]
glmM	produces the essential intermediate for peptidoglycan synthesis, it negatively regulate the activity of DacA	[42]
ybbR	acid stress resistance, cyclase regulator , it is a component of a conserved operon that includes three genes, dacA/ybbR/glmM	[43]

Supplementary Table 4

**Supplementary Table 4. Selectivity Index**			
Compound	MIC (mg/ml)	Highest Non-Cytotoxic Concentration Tested (mg/mL)	SI = Highest Non-Cytotoxic Concentration Tested /MIC
Tropinone	16	256	16
Eucalyptol	20	320	16
Methicillin (µg/ml) + Tropinone (mg/ml)	64 + 4	128 + 256	64
	64 + 2		128
	64 + 0.5		512
	32 + 8		32
	32 + 4		64
	16 + 8		32
Methicillin (µg/ml) + Eucalyptol (mg/ml)	64 + 2.5	128 + 320	128
	64 + 1.25		256
	64 + 0.3		1066
	64 + 0.15		2133
	32 + 10		32
	32 + 5		64
	32 + 0.625		512
	16 + 2.5		128

Supplementary Table 4: Selectivity Index of the selected compounds alone or in combination with Methicillin. MIC, minimum inhibitory concentration; SI, selectivity index calculated as Highest Non-Cytotoxic Concentration Tested/MIC against MRSA.

Supplementary Methods

Computational Drug Discovery

Docking based virtual screening of FDA approved drugs against DacA

The X-ray crystal structure of the target protein with PDB ID: 6GYW (DacA) (Resolution 1.70 Å) of Staphylococcus aureus was downloaded from protein databank (https://www.rcsb.org). Protein chain A was selected and all the non-essential molecules were eliminated from the pdb file. Polar hydrogen (H) atoms were added to the protein structure for proper hydrogen bonding. Partial atomic charges were assigned using Gasteiger charges from the AutoDock tools [Morris et al., 2009]. The crystal structure was then energy minimized by 100 steps of steepest descent and conjugate gradient minimization in chimera [Pettersen et al., 2004] to avoid any steric clashes. The energy minimized cleaned structure was saved in pdb format for docking. FDA approved Drugbank inhibitors were used in this study as drug library for studying antibacterial activity. A total of 10,614 FDA approved drug bank drugs were extracted from PubChem database (https://pubchem.ncbi.nlm.nih.gov) in sdf format. These sdf files were converted to pdbqt format with python script available in Autodock tool utilities [Morris et al., 2009]. A PDBQT file contain four things for docking such as partial charges, Autodock atom types, rotatable bonds for flexibility in the ligand for docking, and the number of torsional degrees of freedom used to evaluate the conformational entropy. After conversion of files, these files were prepared for docking with MGLTools, a virtual screening tool using python script. In virtual screening process all the ligand molecules were separately processed and docked to the receptor, one ligand at a time. After all the docking jobs were completed, each docking log file parsed by extracting the estimated free energy of binding, the results were combined and sorted based on top scoring ligands and saved the consolidated list in a final result. Further, Pymol-2.3.3 software [Bramucci et al., 2012] was utilized for complex analysis and visualization. Schrödinger software (Schrödinger Release 2023-3: Maestro, Schrödinger, LLC, New York, NY, 2023). was used for 2D interaction diagrams of the complexes.

In addition, the screening capability of the docking model was assessed via ROC curve analysis (Triballeau et al., 2005). Briefly, the top 50 ligands were selected for ROC curve analysis, 15 decoy molecules for each ligand were generated using the RDKit toolkit (Landrum, 2016). All active and inactive ligands were subsequently docked to the target protein with AutoDock4, and their binding energies were computed. Each ligand was then assigned a binary label, with active compounds designated as 1 and inactive compounds as 0. Using these classifications, the model's sensitivity and specificity were calculated to assess its ability to distinguish between active and inactive molecules. Sensitivity was defined as TP/(TP + FN), and specificity as TN/(TN + FP). The performance and reliability of the scoring function were further evaluated using the area under the ROC curve (AUC). An AUC value of 0.5 indicates no better than random classification, whereas values approaching 1 reflect strong discriminatory capability.

ADME/toxicity prediction (SwissADME, ProTox-II) and shortlisting top candidates

SMILES format of the selected compounds was used for ADMET properties prediction with pkCSM web server (https://biosig.lab.uq.edu.au/pkcsm/). The results were exported in a tabulated format for Adsorption (Water solubility, Caco2 permeability, Intestinal absorption, Skin Permeability, P-glycoprotein substrate, P-glycoprotein I inhibitor, P-glycoprotein II inhibitor), Distribution (VDss, Fraction unbound, BBB permeability, CNS permeability), Metabolism (CYP2D6 substrate, CYP3A4 substrate, CYP1A2 inhibitior, CYP2C19 inhibitior, CYP2C9 inhibitior, CYP2D6 inhibitior, CYP3A4 inhibitior), Excretion (Total Clearance, Renal OCT2 substrate), and Toxicity (AMES toxicity, Max. tolerated dose, hERG I inhibitor, hERG II inhibitor, Oral Rat Acute Toxicity (LD50), Oral Rat Chronic Toxicity (LOAEL), Hepatotoxicity, Skin Sensitisation, T. Pyriformis toxicity, Minnow toxicity. Comparative analysis identified the favorable ADMET properties for the shortlisted compounds.

Selectivity Index

Selectivity Index (SI) was calculated as described previously (Xu et al., 2026). However, as no measurable cytotoxicity was observed at the highest concentrations tested for either of the drugs or the drug combinations in tis study, precise CC_{50} values could not be determined. Therefore, SI were conservatively estimated using the highest non-cytotoxic concentration tested divided by the effective concentration of each compound in combination assays.
